# Tree-based exploratory identification of predictive biomarkers in non-randomized data

**DOI:** 10.1186/s12874-026-02928-8

**Published:** 2026-06-27

**Authors:** Julia Krzykalla, Maral Saadati, Wiebke Hielscher, Annette Kopp-Schneider, Hartmut Döhner, Axel Benner, Dominic Edelmann

**Affiliations:** 1 Boehringer Ingelheim Pharma GmbH & Co. KG, Biberach, Germany; 2 Division of Biostatistics, German Cancer Research Center (DKFZ), Heidelberg, Germany; 3 Department of Engineering and Management, Technical University of Applied Sciences Mannheim, Mannheim, Germany; 4 Department of Internal Medicine III, Ulm University Hospital, Ulm, Germany

**Keywords:** Predictive factors, Treatment effect modification, PredMOB, Non-randomized data, Confounder adjustment

## Abstract

The idea of “stratified medicine” is an important driver of methodological research on the identification of predictive biomarkers. Most methods proposed so far for this purpose have been developed for the use on randomized data only. However, often data from clinical registries or non-randomized studies are the only available data source. For such data, methods for estimation of the average treatment effect are well established. Research on confounder adjustment when investigating the heterogeneity of treatment effects and the variables responsible for this is usually restricted to regression modelling. In this paper, we demonstrate how the predMOB, a tree-based method that specifically searches for predictive factors, can be combined with common strategies for confounder adjustment. In an extensive simulation study, we show that the proposed strategies allow for the correct identification of predictive factors in the presence of confounding. It is demonstrated that the most robust method is a combination of both covariate adjustment and Inverse Probability of Treatment Weighting (IPTW). Applications to the German Breast Cancer Study Group (GBSG) trial 2 and the Acute Myeloid Leukemia Study Group (AMLSG) 16-10 study illustrate these conclusions.

**Supplementary Information:**

The online version contains supplementary material available at 10.1186/s12874-026-02928-8.

## Introduction

Especially in cancer research, the idea of a therapy that is specifically tailored to the individual patient has been increasingly sought in recent decades. The choice of the best treatment option is usually founded on baseline measures (for example demographics, disease characteristics, laboratory parameters or genetic information) that help determine which treatment is most effective for a given patient. Those variables are called “predictive factors” or “treatment effect modifiers”. The term treatment effect modification expresses that the effect of a specific treatment varies as a function of some baseline feature. In this paper we focus on the search for features that could define strata with differential treatment effects.

Importantly, treatment effect modification applies to a very specific context. A predictive factor for the relationship of a certain treatment on a certain outcome does not necessarily have to be a predictive factor for another treatment as well, even if that treatment targets the same disease; nor does it imply effect modification with respect to an alternative effect measure. Moreover, effect modification is relative to the population as it might be affected by the distribution of a third factor which is neither the treatment nor the outcome. A very common example for such a factor is the ethnicity of a patient [11].

In a statistical regression model, predictive factors are represented by an interaction term of the respective variable with treatment. If only pre-defined variables are to be investigated, simple interaction tests, possibly with a subsequent adjustment for multiple testing, may be sufficient, although they may be compromised by low power [14]. Yet, in situations in which the pathways of the disease itself or the treatment’s mode of action are not fully understood, the number of potential candidates might be large. In these cases, tree-based methods are advantageous for performing an exploratory search across all candidates [26]. Besides being able to handle a large number of variables at the same time, these methods are also very flexible as they do not require prespecification with regard to the functional form of the interaction nor the order of the interaction. Prominent examples of such methods are interaction trees [21], causal forests [1, 24] or Model-based Recursive Partitioning for Subgroup Analysis (MOB) [18]. Most of these methods provide a precise prediction of the individual treatment effect, but ignore interpretability of the resulting tree or forest. That is, factors with exclusively prognostic effects, i.e. affecting the outcome irrespective of any treatment, may be selected as well. Furthermore, simultaneous selection of splitting variable and cut-off, as is done by most methods, favors variables with multiple cut-offs, particularly continuous variables. Krzykalla et al. [13] proposed the predMOB, which, as an extension of MOB is specifically tailored to identify predictive factors.

As the majority of existing tree based methods (except for the causal forests), the predMOB has been developed for randomized settings. In practice, data from randomized trials are not always available, so one has to rely on non-randomized data. Standard statistical methods may yield unsatisfactory results when applied naively to such data due to the presence of confounding. This problem is commonly addressed by the use of adjustment methods. The purpose of this paper is to investigate how predMOB can be used to correctly identify predictive factors based on non-randomized data in an exploratory search. We examine whether adjustment for confounding is necessary and if so, which of the common adjustment strategies (covariate adjustment, Inverse Probability of Treatment Weighting (IPTW)) can achieve an appropriate correction.

In this manuscript, our primary aim is to present adaptations of the predMOB framework for non randomized settings as a proof of concept. In line with the role of a methodological phase II simulation study, our goal here is to provide empirical evidence that the proposed adaptation behaves sensibly in finite samples across a limited range of scenarios, thereby establishing its validity and motivating further research [8]. A comprehensive benchmarking exercise represents an important next step in the methodological development process, but it lies beyond the intended scope of the present work.

Furthermore, a direct comparison with other established tree-based approaches, such as interaction trees [21] or causal forests [1, 24], is not straightforward in the context of this work. As discussed in Krzykalla et al. [13], many existing tree-based methods are primarily designed to estimate heterogeneous treatment effects, with a focus on predictive accuracy rather than on identifying predictive factors in the strict biomarker sense. Their splitting criteria and target estimands therefore differ fundamentally from those of predMOB, which is explicitly constructed to detect treatment–covariate interactions. Moreover, while causal forests estimate conditional average treatment effects, they do not inherently distinguish between prognostic and predictive structure, and interaction trees—although conceptually closer—rely on different modeling assumptions and do not incorporate confounder adjustment in the way required for non-randomized data. Substantial methodological modifications would thus be needed to render these approaches comparable to the framework studied here, and such adaptations lie beyond the scope of the present proof-of-concept investigation.

A note on comparisons of MOB and predMOB: While the MOB framework indeed allows for more flexible specifications - such as including known prognostic factors directly in the model while restricting the splitting variables to predictive candidates - this manuscript does not aim to optimize or extend MOB itself. Our focus is on introducing predMOB extensions for non-randomized settings, and the standard MOB implementation serves as the natural reference because predMOB is derived directly from it. Even with preselected prognostic factors, MOB still tests for instability in both intercept and treatment parameters, so prognostic and predictive effects cannot be fully separated. In contrast, predMOB is explicitly constructed so that every split reflects heterogeneity in the treatment effect. A detailed exploration of alternative MOB specifications would broaden the scope of the paper and is therefore beyond our current aims, but we acknowledge that such options exist and may be useful in applied analyses.

To demonstrate the use of predMOB for detecting predictive factors, we present extensive simulation studies designed to reflect some simple as well as some more complex, real world structures. In particular, our simulation design is motivated by applications in which predictive factors correspond to the presence or absence of genetic alterations or other binary clinical features, therefore we use binary variables. As screening of individual genes is increasingly being replaced by gene panel diagnostics in medical applications [4], we consider it useful to also replicate such a (high-dimensional) gene panel.

To illustrate the use of predMOB we show two real-data examples. The first example is on identification of predictive factors for the use of tamoxifen in women with node-positive breast cancer enrolled in the German Breast Cancer Study Group 2 (GBSG2) study. This trial was set up as a Comprehensive Cohort Study, i.e., part of the patients were randomized while others were treated according to their or their doctor’s decision. The special setup enables us to compare the results from the predMOB applied to the randomized patient set to those from the non-randomized part, obtained by predMOB in combination with various methods for confounder adjustment, thus studying the performance of these methods on a real study data set. The second example is the single-arm, phase 2 trial AMLSG 16-10 of the German-Austrian Acute Myeloid Leukemia Study Group (AMLSG). Here, a comparison with a historical control cohort was performed.

In Methods section, we describe the notation of effect modification. Moreover, the predMOB approach is introduced and its combination with the mentioned adjustment methods is outlined. Simulation study section reports the results of an extensive simulation study, investigating which of the adjustment strategies allows a correct identification of predictive factors in a non-randomized setting. Results of the application to the clinical study data are presented in Application example 1: The GBSG2 trial and Application example 2: The AMLSG 16-10 study sections. The paper closes with a discussion and a short description of computational details.

## Methods

This section briefly introduces effect modification and explains how to quantify it in the presence of confounding within a simple regression model. Subsequently, we describe how predictive factors can be identified using the predMOB [13] and how the adjustment methods mentioned in the first section can be combined with such an approach.

### Effect modification

Consider the following setting where for each patient $i, \ i=1, \ldots , n$, $Y_i$ is the outcome of interest and $T_i$ the administered treatment ($T_i=1$ if the patient received the investigational treatment and $T_i=0$ for standard of care). The effect of treatment on outcome is modelled by a generalized linear model with $\mu = \mathbb {E(Y)}$ and $g(\mu ) = \alpha + \beta _T T$, where *g* denotes the link function.

Effect modification means that the treatment effect differs across strata of an effect modifier *M*. A common way of assessing whether *M* is a treatment effect modifier is to test whether $\gamma$ is non-zero in the following model

1 $$\begin{aligned} g(\mu ) = &\alpha + \beta _T T + \beta _M M \\&+ \beta _C^t \textbf{C} + \gamma MT, \end{aligned}$$

where $\textbf{C}$ denotes a random vector of relevant confounders.

However, the functional form specified in (1) may be too restrictive. An alternative approach is to fit a propensity score model for the probability of treatment as function of the confounders and the treatment effect modifier, that is,

$$\begin{aligned} P(T = 1|M,\textbf{C}) = e(M,\textbf{C}). \end{aligned}$$

Each observation *i* is then weighted by the inverse of the estimated probability of receiving treatment, $\widehat{e}(M_i,\textbf{C}_i)^{-1}$.

An alternative to propensity score weighting is propensity score matching. Since both approaches give very similar results, we focus on propensity score weighting as this does not discard unmatched observations.

In practice, IPTW and regression adjustment for confounders are often combined providing procedures that remain consistent if either the propensity score model or the outcome model is correctly specified.

### Identification of predictive factors using the predMOB

As mentioned in the introduction, the MOB [18] is one of the popular examples of tree-based methods for subgroup identification. The method starts with a base model that only includes treatment

2 $$\begin{aligned} g(\mu )=a+b \cdot T \end{aligned}$$

and then recursively splits the population in two subgroups whenever the coefficients of this base model, either the intercept *a* or the coefficient for the treatment effect *b*, are “instable” with respect to one of the potential splitting variables and thus differential across the resulting subgroups. This instability of the model parameters is assessed using the M-fluctuation test [27]. However, the method has a major drawback with regard to the identification of predictive factors as the splitting rule allows splits for prognostic factors as well, as these are factors responsible for “instabilities” in the intercept. Consequently, it is not possible to distinguish between prognostic and predictive factors using this approach. To overcome this problem and focus on predictive factors in the construction of the tree, Krzykalla et al. [13] proposed to reparametrize the base model using effect coding instead of dummy coding for the treatment variable ($T^*= 1 \text { for active treatment and } T^*= -1 \text { for control}$). Assuming balanced treatment assignment ($\mathbb {P}(T^*=1)=\mathbb {P}(T^*=-1)=\frac{1}{2}$), a valid working model consists only of the treatment effect without intercept

3 $$\begin{aligned} g(\mu ) = b \cdot T^*/2 \end{aligned}$$

such that each split can be attributed to a predictive factor, while the estimate $\hat{b}$ for the treatment effect in model (3) is the same as the estimate for *b* in model (2). In order to decide whether a variable is a predictive factor, multiple predMOB trees are grown - each on a subsample of the original data - and the importance of all potential predictive factors on the construction of this random forest are assessed using variable importance measures such as the permutation importance [2]. Permutation importance measures the drop in prediction accuracy for a variable of interest caused by breaking its relationship with the outcome via permutation. The use of subsampling (without replacement) rather than bootstrapping (with replacement) is especially preferable when using random forests in combination with permutation importance to avoid the introduction of bias [20].

The proposed reparametrization of the base model for predMOB enables to focus on predictive factors and thus reduces the number of variables that are incorrectly identified due to a purely prognostic effect. The modification is inspired by the modified covariates approach for (generalized) linear regression models of Tian et al. [22].

Note that an offset is used in the unadjusted and IPTW predMOB model fit to take into account possible imbalances between treatment groups and to stabilize the resulting predMOB model, which does not include an intercept. The offset is calculated as the global mean of the outcome *Y* transformed using the respective link function.

### Confounder adjustment when using predMOB

Combining the adjustment methods for regression models with tree-based methods or methods based on random forests is not straightforward. The analogue of covariate adjustment in a (generalized) linear regression model for a MOB tree is a so-called partially additive (generalized) linear model tree (palmtree) [17]. The fit of a MOB tree is determined by the regression coefficients for intercept and treatment effect in each of the leaf nodes. It can hence be expressed via

$$\begin{aligned} g(\mu ) = I(s=1)&\boldsymbol{\tilde{x}}^T\boldsymbol{\tilde{\beta }}_1 + I(s=2)\boldsymbol{\tilde{x}}^T\boldsymbol{\tilde{\beta }}_2 \\& + \ldots = \boldsymbol{\tilde{x}}^T\boldsymbol{\tilde{\beta }}(s) \end{aligned}$$

where $s=1,2,\ldots$ represents the assignment to the subgroup that corresponds to the leaf node in the tree and $\boldsymbol{\tilde{x}}^T = c(1,T)$. This means that all model parameters $(\boldsymbol{\tilde{\beta }}^T_1, \boldsymbol{\tilde{\beta }}^T_2, \ldots )$ are depending on the structure of the tree. In contrast to this, palmtrees allow the inclusion of factors with a global effect ($\boldsymbol{x_F}$) on the outcome, that is, their effects $\boldsymbol{\gamma }$ do not depend on the tree structure:

$$\begin{aligned} g(\mu ) = \boldsymbol{\tilde{x}}^T\boldsymbol{\tilde{\beta }}(s) + \boldsymbol{x_F}^T\boldsymbol{\gamma }. \end{aligned}$$

The only difference between MOB and predMOB is the parametrization of the base model, i.e. $\boldsymbol{\tilde{x}}^T = T^*$. Confounders can be introduced into a predMOB tree analogously as factors with a global effect on the outcome using the palmtree approach. No offset is required for predMOB when using palmtrees for covariate adjustment as the global effect on the outcome is already included in the model.

Combining the predMOB with IPTW is carried out by calculating the weights based on a propensity score model as described in Effect modification section. The algorithm for growing the predMOB tree is then run on the pseudo-randomized population obtained by applying those weights to the data. That is, the weights are used both in fitting the base model as well as in computing the M-fluctuation test statistic for the splitting decision within every node. Following the idea of a doubly robust adjustment, predMOB can also be combined with both covariate adjustment and IPTW. This implies growing a palmtree on a pseudo-randomized population obtained by applying the inverse of the probability of being treated with the actual treatment as case weights to each observation. In doing so, predMOB still, in the long run, identifies the true predictive factors as long as at least one model is correct, the propensity model or the outcome model.

## Simulation study

We investigate whether predMOB in combination with common adjustment methods (described in the previous section) yields reliable results when applied to non-randomized data. The adjustment methods explored in this simulation study are covariate adjustment, IPTW, and their combination. The calculation of the weights is done once for the entire data set and passed to the subsamples for the construction of the single trees. In order to reflect a real application when the true set of confounders is not known with certainty, all biomarkers are used for fitting the propensity score model.

The evaluation is made with regard to the correct identification of the predictive factor(s). We consider a variable to be a potentially predictive factor if it shows high variable importance.

Throughout the simulation studies, every forest is an ensemble of 250 trees and every tree is fit to a subset of the original data by subsampling of $63.2 \%$ of all observations (to mimic the amount of information in a bootstrap sample drawn with replacement, cf. Strobl et al. [20]). For the splitting decision, the raw p-values of the M-fluctuation test (without adjustment for multiplicity) are compared against a significance level of $\alpha =0.05$ in order not to be too restrictive. The permutation importance is calculated for each tree on the 36.8% of the data that was not sampled. The resulting overall permutation importance from the forest is the average over all 250 trees. All results are based on 500 simulation runs.

### Identification of predictive factors

For the evaluation of predMOB in identifying the true predictive factor(s), data has been generated according to the following data-generating process and outcome generating models using the simstudy package in R:

- Ten independent and identically distributed biomarkers $X_1, \ldots , X_{10}$; $X_i\sim B(1,0.5)$, transformed from $\{0,1\}$ to $\{-1,1\}$,
- Treatment variable $T \sim B(1, p)$, depending on biomarkers $X_1, \ldots , X_7$ via the logistic regression model $$\begin{aligned}\text {logit}(p)&= \beta _0+\log (1.1)X_1 - \log (1.2)X_2 \\& + \log (1.3)X_3 - \log (1.1)X_4 + \log (1.2)X_5 \\& - \log (1.3)X_6 + \log (1.4)X_7,\end{aligned}$$ with $\beta _0$ being chosen so that $p=0.5$,
- Binary distributed outcome variable $Y \sim B(1, \pi )$ with $\text {logit}(\pi ) = \mu$ as defined in Table 1,
- Sample size $n=1000$ (resulting in 632 observations per subsample).

**Table 1:** Parameter configurations for the simulation study concerning the identification of predictive factors. In all simulation scenarios we use the same base formula given as $0.25 T + 0.1X_4 + 0.15X_5 + 0.2X_6 + 0.25X_7 + 0.2X_8 + 0.1X_9 + 0.15 X_{10}$

Description	Parameter configuration
0 : Null scenario	*μ* = 0
A : Prognostic effects only	*μ* = base formula
B: *X*_10_ is prognostic and predictive	*μ* = base formula + 0.5 *X*_10_ · *T*
C: *X*_10_ is predictive only	*μ* = base formula − 0.15 *X*_10_ + 0.5 *X*_10_ · *T*
D: *X*_3_ is predictive only	*μ* = base formula + 0.5 *X*_3_ · *T*
E: *X*_7_ is prognostic and predictive	*μ* = base formula + 0.5 *X*_7_ · *T*
F: same as scenario B, but smaller interaction effects	*μ* = −0.125 · *T* + base formula + 0.25 *X*_10_ · *T*
G: Correlated biomarkers	G.1: same as scenario B, but Corr (*X*_7_, *X*_10_) = −0.7
	G.2: same as scenario G.1, but *X*_10_ is an unobserved confounder
H: Multiple predictive biomarkers	H.1 *μ* = base formula − 0.5 *X*_9_ · *T* − 1 *X*_10_ · *T*
	H.2 *μ* = base formula + 0.2 *X*_9_ − 0.5 *X*_9_ · *T* − 0.5 *X*_10_ · *T*
I: Higher order predictive pattern (*n* = 2500)	I.1 *μ* = base formula − 0.1 *X*_10_ + 0.5 *X*_9_ · *X*_10_ · T
	I.2 *μ* = base formula − 0.1 *X*_10_ + 0.5 *X*_3_ · *X*_9_ · *X*_10_ · T; Corr (*X*_7_, *X*_9_, *X*_10_) = 0.7
	I.3 same as I.2 but *X*_10_ is an unobserved confounder
J: Higher dimensions	J.1 scenario C with added nuisance variables *V*_1_, . . . , *V*_20_, V_*i*_ ∼ Bin (1, 0.5)
	J.2 *V*_1_, . . . , *V*_100_, *V*_1:5_ ∼ Bin (1, 0.5), *V*_5:20_ ∼ Bin (1, 0.25), *V*_80:100_ ∼ Bin (1, 0.05)

Note, that true predictive factors are implemented by an interaction of the respective variable with treatment in the outcome generating model (Table 1). The number of potential splitting variables is set to 10, i.e. all biomarkers are eligible for each split, except in scenarios J.1 and J.2.

We restricted the simulations to binary covariates, as our goal is to derive treatment recommendations based on input data that can be formally described in terms of interactions with binary variables.

We acknowledge that the propensity score model cannot generally be assumed to represent the true underlying data-generating process in real-life applications. Nonetheless, we view it as a practical and effective tool for addressing imbalances between treatment arms in non-randomized studies. To illustrate the interdependencies between selected covariates and treatment Figure S.21 in Supplement Section S7 shows the distribution of covariates by treatment arm averaged over 500 simulated data sets resulting from the data generating process mentioned above.

**Fig. 1:**
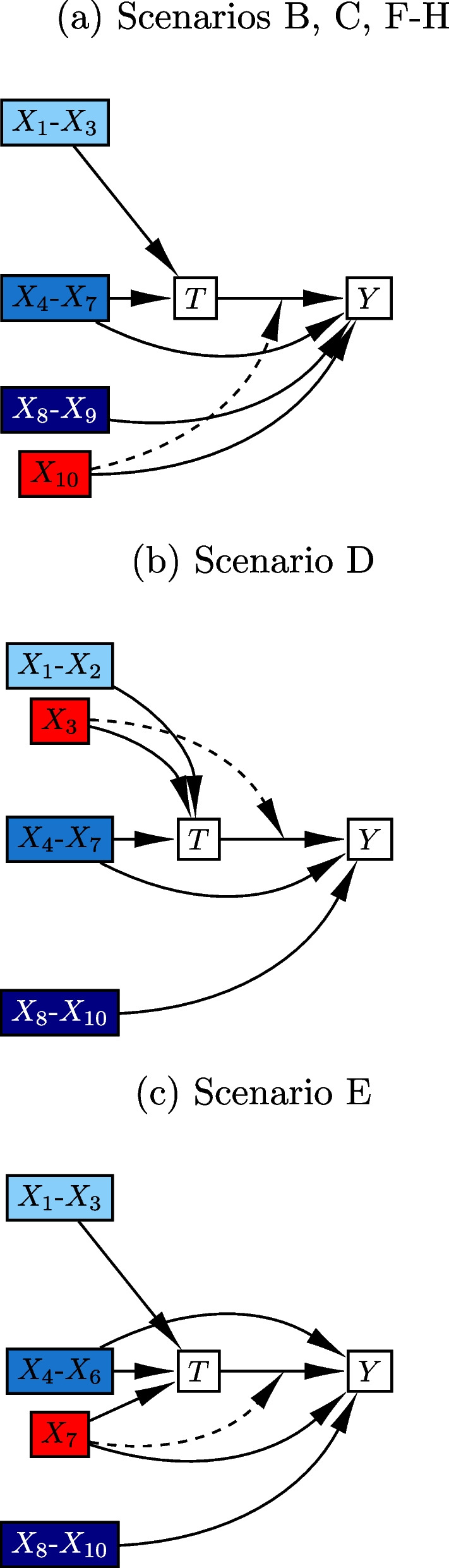
Graphical illustration of the simulation scenarios. Effect-modification is marked as dashed arrows on the edge representing the treatment effect. Variables that only affect treatment assignment are shown in light blue, true confounders (associated with treatment assignment and outcome) in medium blue and factors only associated with outcome in dark blue; predictive factors are highlighted in red

**Fig. 2:**
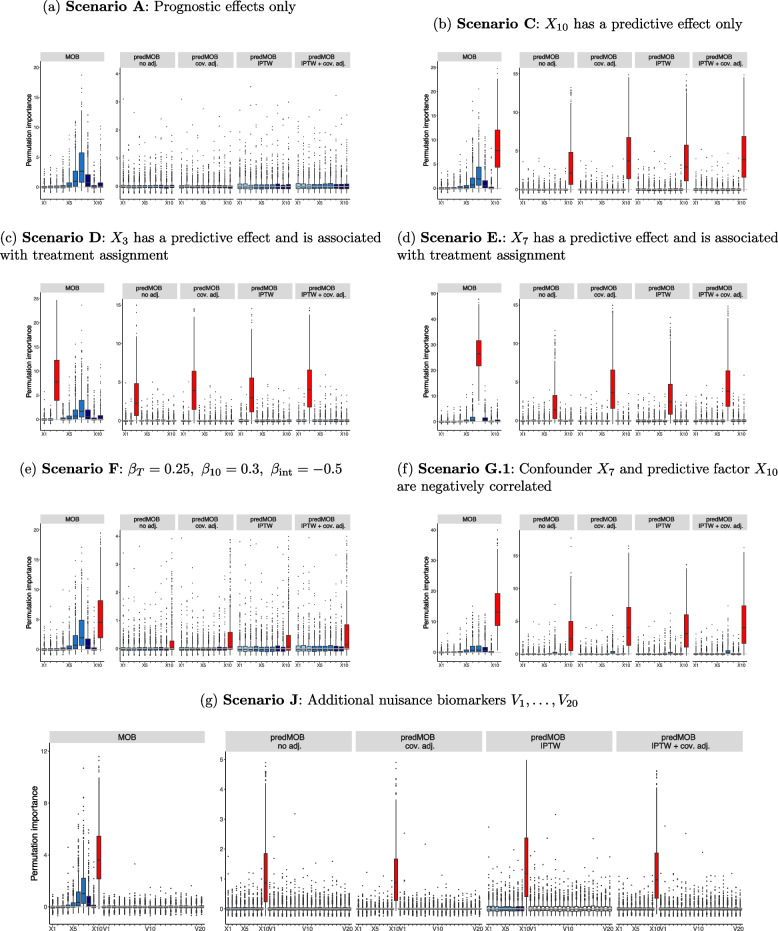
Permutation importance for the MOB and predMOB approaches in combination with various adjustment methods. Instrumental variables are shown in light blue, true confounders in medium blue and factors only associated with outcome in dark blue. The boxplot for a true predictive factor is highlighted in red. Interpretation note: Permutation importance values close to zero indicate that a variable does not have a predictive effect. Accordingly, the figures are scaled to emphasize whether any variable shows a meaningful deviation from zero. Small random fluctuations around zero are not substantively informative, whereas true predictive factors appear as boxplots clearly shifted above the zero line

Scenarios 0 and A are designed to serve as reference scenarios with none of the biomarkers being predictive factors, whereas in scenarios B and C, biomarker $X_{10}$ is the true predictive factor. Apart from that, it is not associated with the treatment assignment. In contrast to this, in scenario D, the predictive factor $X_{3}$ is an instrumental variable, that is, it is associated with the treatment assignment, but has no main effect on outcome. Finally, in scenario E, the confounding variable $X_{7}$ (associated with the treatment assignment as well as with the outcome) is chosen as the true predictive factor. Scenarios F, G, I and J describe more complex settings in order to investigate how results are affected by design modifications such as non-zero correlations between the true predictive factor and other prognostic factors, small interaction effects (“predictive effects”) or an increasing number of candidate biomarkers. Furthermore, scenarios H and I add complexity in the effect modification pattern: either two or more biomarkers are altering the treatment effect independently or act in combination. An illustration of the simulation scenarios is given in Fig. 1. Since the results for some of the scenarios are very similar, only parts of the simulation study are discussed in detail in the main part of the paper (see Fig. 2). The results of the remaining scenarios are presented in the Supplement S1.

In the figures, the variable importance scores over all simulation runs are summarized in boxplots. A colour code is used to distinguish the biomarkers according to their function: instrumental variables are shown in light blue, true confounders in medium blue and factors only associated with outcome are coloured in dark blue. The boxplot for a true predictive factor is highlighted in red. To aid interpretation, it is important to note that the purpose of the permutation-importance plots is to identify variables with values meaningfully above zero. Small variations around zero are expected under the null and should be interpreted as the absence of a predictive (in the case of MOB also potentially a prognostic) effect; visual differences among these near-zero values are therefore not substantively relevant. The figures are scaled accordingly, highlighting the key signal of interest—namely, when a true predictive factor is detected, which is visible as a boxplot clearly shifted above the zero line. Furthermore, the assessment of variable importance scores is meaningful in comparison between biomarkers, i.e., when evaluating variable importance scores of one predMOB run, a high score of one biomarker compared to other biomarkers will be interpreted as indication of the biomarker being a predictive factor. Hence the ranking of variable importance scores for each simulation run provides additional information as to whether the true predictive factor is consistently identified. Ranking results are provided in Supplement Section S6.

#### Scenarios 0 & A:

In scenario 0 none of the biomarkers is associated with the outcome (see Supplement S1) Thus there are only instrumental variables and random variables that are neither associated with the treatment assignment nor with outcome. The permutation importance of all approaches fluctuates around zero regardless for all variables of whether and which adjustment strategy was used. Variability is also comparable across all analysis strategies. In contrast to this, scenario A includes three types of variables: instrumental variables, true confounders and variables with a prognostic effect on the outcome (results depicted in Fig. 2a). The MOB approach is able to detect variables associated with outcome (as expected with higher permutation importance for variables with larger effects, $X_{4}$ and $X_{9}$ seem too small to detect). As still none of the variables are predictive factors, all permutation importance measures for predMOB methods should be equal to zero, like in scenario 0. For all predMOB analysis strategies the permutation importance for all variables fluctuates randomly around zero. The inclusion of IPTW and covariate adjustment shows no advantage over the unadjusted approach.

#### Scenario B & C:

In these two scenarios, biomarker $X_{10}$ has been generated with a large treatment effect modifying effect. Figure 2b shows the results for scenario C. Since the plots for scenario B is very similar, the results are shown in the Supplement S1. Like in scenario A, permutation importance values for the predMOB approaches are observed fluctuating randomly around 0 for all variables that are not predictive factors. However, $X_{10}$ is the only variable with clearly positive permutation importance values and thus the only variable identified as a predictive factor, irrespective of the adjustment. MOB also detects $X_{10}$ as a relevant variable, but with the usual caveat that it cannot distinguish between prognostic and predictive factors.

#### Scenario D & E:

In contrast to the scenarios B & C, the true predictive factor in scenarios D and E is also associated with the treatment assignment. In both cases, MOB is able to clearly detect the treatment effect modifying variable $X_{3}/X_{7}$ along with other prognostic variables $X_{4}$ to $X_{10}$ depending on the effect size. Furthermore, predMOB with covariate adjustment outperforms the not-adjusted version, as it yields the strongest superiority of $X_{3}/X_{7}$ over all non-predictive factors in terms of higher permutation importance. The use of IPTW in addition to covariate adjustment does not provide any further improvement in these scenarios.

#### Scenario F:

Scenario F basically describes the same setting as scenario C, but with smaller effect sizes (cf. Fig. 2e) and hence, a lower chance to detect the true predictive factor. As a result, $X_{10}$ is no longer consistently identified by predMOB if no adjustment or IPTW is applied.

#### Scenario G:

Here we examine the implications of the true predictive factor being negatively correlated with a confounder variable. To generate an extreme scenario, the strongest prognostic factor $X_7$ was chosen to be correlated with the true predictive factor $X_{10}$. In scenario G.1 $X_7$ and $X_{10}$ are negatively correlated and both variables are available in the data set, as opposed to scenario G.2 where variable $X_{10}$ is an unmeasured confounder (i.e. not included in the set of variables to be tested as a predictive factor).

Results for scenario G.1 are visualized in Fig. 2f, where we observe that predMOB methods correctly identify $X_{10}$ as a predictive factor. When applying covariate adjustment with and without IPTW the permutation importance of X7 is slightly increased and thus might be falsely detected as predictive factor.

In scenario G.2 (see Supplement S1) $X_{10}$ is actually a predictive factor, but is considered an unmeasured confounder. In this case, we would hope to detect $X_7$ as a kind of surrogate. Here MOB and predMOB methods are able to detect $X_7$ albeit with a rather low permutation importance that makes it hard to distinguish from non-relevant variables at times.

#### Scenario H & I:

Although there is more than one predictive factor in scenarios H & I.1, the results are very similar to the scenarios B & C. Both predictive factors are correctly identified by predMOB methods no matter which adjustment strategy is used, and covariate adjustment is generally more effective than the non-adjusted version (especially in scenario H.1). In the more complex scenarios I.2 and I.3 it becomes increasingly harder to distinguish predictive factors, shown by the corresponding red box plots being closer to zero. As these conclusions are very much in line with the other scenarios, results are only shown in the Supplement S1.

#### Scenario J:

The results for scenario J.1 are essentially the same as for scenario C.1: $X_{10}$ can be correctly identified by predMOB approaches as the only predictive factor. We observe less fluctuation (variability) for the other variables if only covariate adjustment is applied in the scenario. No adjustment and IPTW seem to have additional variability in the permutation importance with more noise variables. In scenario J.2 we observe that a large set of nuisance variables makes it more difficult for predMOB to identify the predictive factor $X_{10}$ and that the corresponding box plot for the permutation importance is only slightly higher than for non-predictive factors.

In Supplement Section S6 we present an alternative depiction of the simulation results using ranking methods in the spirit of Wiesenfarth et al. [25]. We observe that true predictive factors are most often ranked 1 with all predMOB approaches (scenarios B-G.1, J.1), higher order interactions of predictive factors are often top-ranked (scenarios H.1, H.2, I). Variables that are not predictive factors show an even distribution to the lower ranks. The differences between the predMOB adjustment methods are less pronounced compared to the evaluation of the actual value of the permutation importance score.

In summary, as is expected, the MOB approach is able to detect prognostic and predictive factors, but fails to distinguish between them. All predMOB approaches detect predictive factors by design and were able to identify the respective variables in all settings assigning them consistently the highest rank (Supplement Section S6). We observe a small improvement in terms of higher permutation importance when using covariate adjustment compared to the non-adjusted predMOB. Not surprisingly, combining covariate adjustment with IPTW shows no advantage over covariate adjustment alone in the scenarios investigated here. This is due to the fact that the simulated scenarios were simple enough (linear predictors for both treatment assignment and outcome) that the proposed methods could easily fit the data. In the Supplement Section S2 we present simulation results from settings with non-linear association for treatment assignment, where the IPTW approach and non-adjusted predMOB perform worse, thereby demonstrating the need for covariate adjustment in non-randomized settings. Accordingly, in Supplement Section S3, we show results for the situation of a nonlinear dependency of the outcome on predictors, where the IPTW approach performs better than the model that uses only covariate adjustment ignoring the nonlinear association.

## Application example 1: The GBSG2 trial

An important criterion for the treatment decision in breast cancer patients is the hormone receptor status of estrogen and progesterone. Cancer cells with a positive hormone receptor status are known to be responsive to therapies that lower the hormone levels or prevent the fostering of cancer cells by the respective hormone.

Between 1984 and 1989, 720 node-positive breast cancer patients entered the German Breast Cancer Study Group (GBSG) trial 2, a Comprehensive Cohort Study [16]. The study medication consisted of chemotherapy with or without the additional use of the estrogen-receptor modulator tamoxifen. Upon trial entry, patients could decide whether they agree to be randomized to one of four arms (3 versus 6 cycles of chemotherapy, each with or without tamoxifen) or whether they themselves or the treating physician shall be responsible for the treatment decision. From December 1986 on, premenopausal patients were randomized only with respect to the chemotherapy regimen while randomization with respect to tamoxifen was no longer performed for those patients [16]. In the following, we use ’randomized patients’ to refer to those who have been randomized with respect to tamoxifen.

Our analysis is based on all 720 patients and missing covariate information is imputed using Multivariate Imputation by Chained Equations (mice) with 10 imputations [23]. Furthermore, since predMOB has so far only been developed for normal and binary endpoints, Relapse-free survival (RFS) times are converted to a binary endpoint using a cut-off of two years. Patients experiencing an event related to RFS within the first two years after study start shall be separated from patients who do not experience such an event within this time frame or at all. This leads to 179 patients having a RFS event and 514 patients without event. For the 27 patients censored within the first two years, the RFS status at 2 years cannot be determined and so these patients were not considered for the analysis.

We acknowledge that the use of survival methods would be more appropriate for the RFS endpoint, but the extension of predMOB for survival endpoints is still an open research topic. Nevertheless, we choose to analyze this data set due to the fact that it contains a randomized as well as a non-randomized part, which is very well suited for the overall research questions of this methodical paper. Therefore, we apply the proposed methods to a dichotomized version of the outcome with the goal of identifying predictive factors in the randomized and non-randomized cohort. Thus we grow one forest on each of the two subpopulations. Following Schmoor et al. [16], we include the following variables in the models: menopausal status pre/post (menostat), tumor size categories (tsize.cat), tumor grade I - III (tgrade), number of affected lymph nodes in categories (pnodes.cat), progesterone receptor status (progrec) and estrogen receptor status (estrec) in fmol/l) for treatment with tamoxifen as candidates in our analysis. Variables with high variable importance scores in the resulting forest are considered to be potentially predictive factors.

Descriptive statistics for the two subpopulations by hormonal therapy yes/no are tabulated in Supplement Section S4. For better comparability, patients randomized with respect to chemotherapy only will not be considered for the analysis. Since these 100 patients are all postmenopausal, adding them to the non-randomized patients would further increase the age difference between the two subpopulations.

The aforementioned variables are used for the propensity score model as well as the prognostic model for the 2-year RFS.

As an initial approach we present the results from a simple multivariable model including all possible interactions with treatment (Supplement S4). In the randomized cohort we observe number of positive lymph nodes and tumor grade as prognostic variables but there is no significant interactions with treatment. Whereas in the non-randomized cohort progesterone receptor status has a significant interaction p-value and may be further explored as a possible predictive factor.

**Fig. 3:**
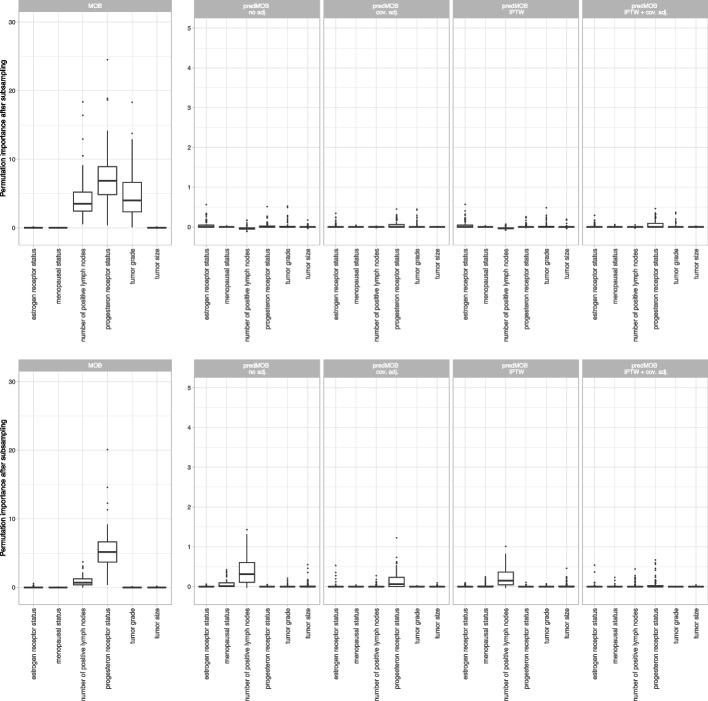
Variable importance for MOB and predMOB in combination with common methods for confounder adjustment based on the set of randomized (top panel) and non-randomized patients (bottom panel) in the GBSG2 study, when restricting to randomization with regard to tamoxifen. In the randomized set no predictive effects are found by the predMOB methods indicating that the variables identified by MOB (number of positive lymph nodes, progesterone receptor status and tumor grade) have only prognostic relevance. We observe similar findings in the non-randomized set for predMOB with IPTW and covariate adjustment

To assess the variability of the permutation importance scores, we generate $B = 100$ subsamples by drawing 80% of observations without replacement and apply the proposed procedures to each of the resulting 100 subsampling datasets. The outputs reported in the following are based on forests grown on each data set split into a $63.2\%$ subset for model fit and the remaining $36.8\%$ for independent variable importance calculation. The number of candidate variables for each split is chosen to be $\lceil {\sqrt{\#variables}}\rceil = 3$. More precisely, forests of 500 trees are grown based on each of 10 imputed data sets, and then aggregated into one large forest for which we calculate the variable importance measure. Variable importance measures for the subsamples are presented as 20% trimmed means (for more robust results) averaged over all 100 subsamples and displayed in box plots (see results in Fig. 3 top panel for randomized and bottom panel for the non-randomized cohort).

In the randomized patient cohort MOB identifies three variables (number of positive lymph nodes, progesterone receptor status and tumor grade) as possibly prognostic or predictive factors. Further inspection via predMOB methods shows that these variables are unlikely to have a predictive effect.

Also in the non-randomized patient set, MOB detects number of positive lymph nodes and progesterone receptor status. Applying predMOB without any adjustment shows a slight increase in permutation importance for the number of positive lymph nodes, but this is attenuated after covariate adjustment and IPTW.

When looking at the ranking of the permutation importance (see Supplement S4) we draw similar conclusions with respect to the lack of predictive factors in both the randomized and non-randomized cohort. It is interesting to note that many variables are given rank 6 in the non-randomized cohort, indicating that no variables were chosen at all for any split in a large number of predMOB trees.

In summary, we were able to detect similar prognostic variables in the randomized and non-randomized cohorts for 2-year RFS. However, no predictive factors were found.

## Application example 2: The AMLSG 16-10 study

The German-Austrian Acute Myeloid Leukemia Study Group (AMLSG) 16-10 study (*n* = 440) was conducted as a single-arm, phase 2 trial for adult patients with acute myeloid leukemia (AML) and fms-related tyrosine kinase 3 (*FLT3*) internal tandem duplication (ITD) to evaluate treatment with midostaurin in addition to intensive chemotherapy in comparison to a historical cohort of patients from 5 prior AMLSG trials who had not been treated with midostaurin, see Döhner et al. [3]. The AMLSG 16-10 trial is registered at clinicaltrialsregistry.eu as Eudra-CT number 2011–003168-63 and at clinicaltrials.gov as NCT01477606.

Patients aged 18 to 70 years with newly diagnosed AML with *FLT3*-ITD and considered fit for intensive chemotherapy were eligible for the AMLSG 16-10 trial. Diagnoses included de novo AML, secondary AML, and therapy-related AML. The historical control cohort consisted of 415 patients aged 18 to 70 years with newly diagnosed AML with *FLT3*-ITD who had received intensive chemotherapy within 5 AMLSG trials conducted between 1993 and 2009.

In this example we consider the binary outcome “response to induction therapy” (defined as complete remission (CR) or complete remission with incomplete hematologic recovery (CRi)) as the analysis endpoint. An overview of patient baseline characteristics of the AMLSG 16-10 study and the historical control cohort is presented in Döhner et al. [3].

In this analysis our goal is to identify predictive factors for treatment with midostaurin. Supplementary Section S5 shows the logistic regression model for endpoint “response to induction therapy” including all main effects and treatment interactions of established clinical variables. A significant interaction between sex and treatment is observed.

**Fig. 4:**
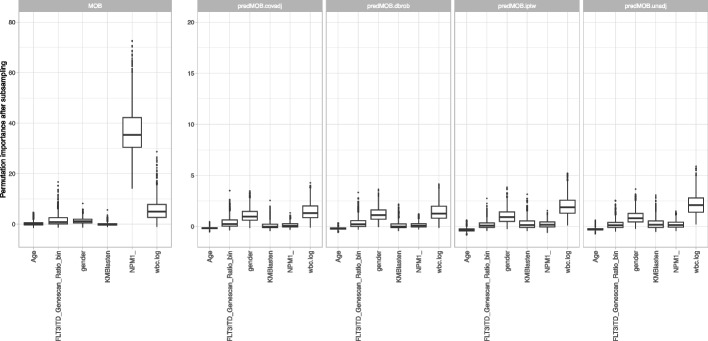
Variable importance for MOB and predMOB in combination with common methods for confounder adjustment for the AMLSG 16-10 study endpoint “response to induction therapy”. MOB identifies *NPM1*, white blood cell count and *FLT3*-ITD mutant-to-wild-type allelic ratio (AR), which are clinically established prognostic factors. The predMOB results with IPTW and covariate adjustment indicate that white blood cell count and sex might be predictive factors for an effect of midostaurin treatment

Figure 4 depicts the permutation importance subsampling results for the tree-based methods (B = 100 subsamples, 500 trees per forest, 10 imputed data sets). MOB identifies the prognostic markers *NPM1* mutation status and mutant-to-wild-type *FLT3*-ITD allelic ratio (AR) as well as white blood cell count. Using predMOB we see some indication that white blood cell count and sex may be predictive factors for treatment with midostaurin. The results between the different predMOB methods are comparable with and without covariate adjustment and IPTW.

These findings are in line with the ranking of permutation importance of the variables (Supplementary Section S6.2), where we observe white blood cell count and sex most often ranked 1 and 2 with all predMOB approaches, indicating that these variables are possible predictive factors.

## Discussion

Since data from a randomized treatment comparison is not always available, the idea of basing the search for predictive factors on data from non-randomized studies or registry data seems reasonable. While the literature on estimating average treatment effects is steadily growing, research on identifying predictive factors from non-randomized data remains sparse. In this work, we systematically investigate the potential issues that arises with the unadjusted application of a tree-based method for the identification of predictive factors, the predMOB, to non-randomized data and compare common adjustment strategies with respect to the achieved correction.

Concerning the identification of the predictive factors, all methods show a similar performance for the simulations in the main paper. However, simulations in the supplementary material show that covariate adjustment and IPTW alone can fail in more complex scenarios, whereas the combination of both approaches provides appropriate confounder adjustment throughout all our scenarios. Therefore, we recommend combining covariate adjustment with IPTW for practical applications, as this offers advantages in more complex situations.

We examined the case of including additional random variables that have no effect on the outcome and showed that this does not significantly influence the results. However, we have kept the number of potential confounding factors in the simulations within a low to moderate range that can still be managed with the proposed adjustment strategies. More complex methods that are able to handle high-dimensional sets of confounders are discussed elsewhere [5, 7, 12].

When it comes to the interpretation of the results, the application examples show that a (relatively) large variable importance alone does not provide a definitive decision of whether a factor is truly predictive or not. Variable importance only allows a ranking of the variables according to their contribution in the modelling of the tree.

Further research is also needed to make predMOB applicable to time-to-event data. Since the approach requires a fully parameterized model, one possibility would be to use a Weibull model as the base model. Alternatively, pseudo-values could be used to circumvent the difficulties arising from censored data.

In this manuscript, our primary aim was to present adaptations of the predMOB framework for non-randomized settings as a proof of concept. Specifically, we focused on demonstrating the feasibility of integrating confounder adjustment and inverse probability of treatment weighting into a method originally developed for randomized trials. An empirical comparison for benchmarking predMOB against established tree based approaches - such as causal forests or interaction trees - would be an important next step.

## Computational details

All computations and analyses have been implemented in R, version 4.4.1 [15] together with the packages partykit (version 1.2–23) [10] and model4you (version 0.9–8) [19]. For the simulation study, data has been generated using the simstudy package (version 0.8.1) [6]. Data of the GBSG2 study are partly available in the TH.data package [9].

## Supplementary Information

Supplementary Material 1.

## Data Availability

Data for simulation scenarios was generated as described in the manuscript. Clinical trials data from the application examples are partially available in R packages and partially confidential (these were provided to us by the trial statisticians upon request).
