# Supplementary material for: Tree-based exploratory identification of predictive biomarkers in non-randomized data
Source: BMC Med Res Methodol. 2026 Jun 27;26:144. doi: 10.1186/s12874-026-02928-8 (PMC13312558; doi:10.1186/s12874-026-02928-8)
Supplement: Supplementary file 1 — Supplementary Material 1. [file 12874_2026_2928_MOESM1_ESM.pdf]

Supplementary Material

S 1 Simulation results for the identification of predictive factors: additional scenarios from Table 1

We show the permutation importance results for MOB and predMOB in combination with various adjustment methods in simulation settings from Table 1 that were not presented in the main manuscript. As in Figure 2 instrumental variables are shown in light blue, true confounders in medium blue and factors only associated with outcome in dark blue. The boxplot for a true predictive factor is highlighted in red.

Fig. S.1: **Scenario 0**: Null scenario.

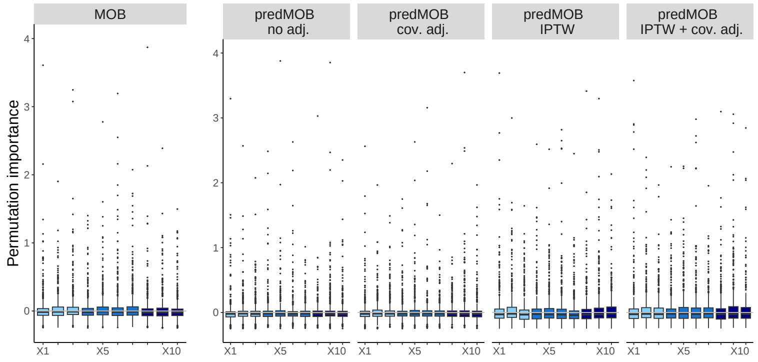

Fig. S.2: **Scenario B**: X_{10} has both a prognostic and a quantitative predictive effect

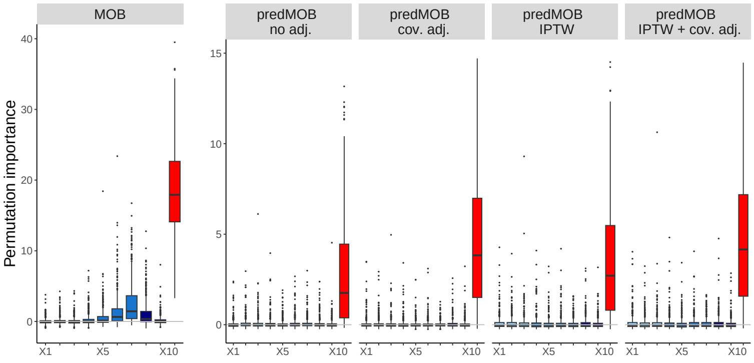

Fig. S.3: **Scenario G2**: Confounding variable X_7 and predictive factor X_{10} are negatively correlated, however, X_{10} is not observed. Note: X_7 is colored red even though it is not a predictive factor, but in this scenario it serves as a kind of surrogate, because it is correlated with an (unobserved) predictive factor.

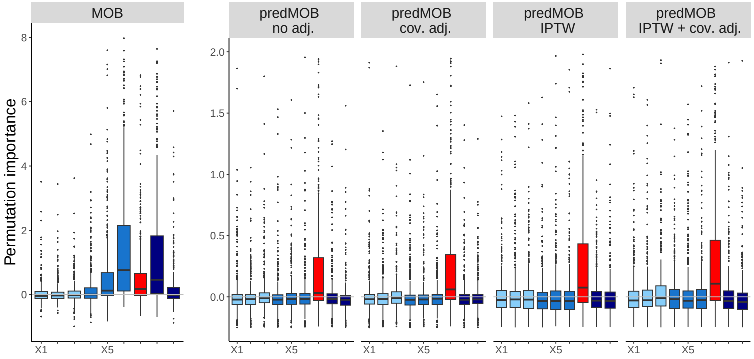

Fig. S.4: **Scenario H.1**: X_9 and X_{10} both predictive only with predictive effect of X_9 smaller than that of X_{10} (cf. Table 1).

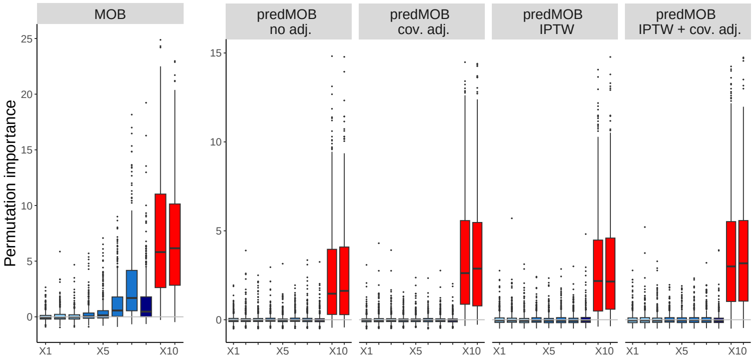

Fig. S.5: **Scenario H.2**: X_9 and X_{10} both prognostic and predictive with different effect sizes (cf. Table 1).

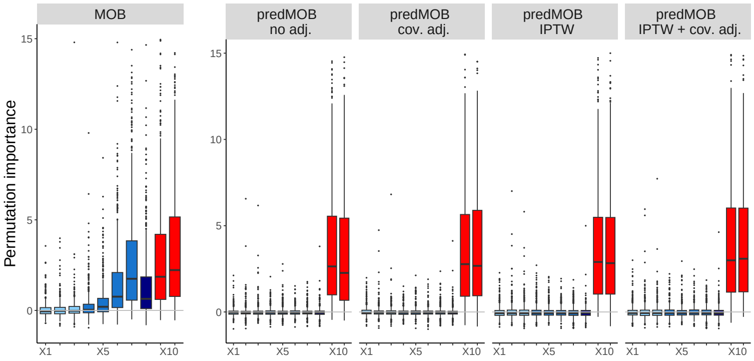

Fig. S.6: **Scenario I1**: Higher order predictive pattern with three-way interaction of X_9, X_{10} and treatment.

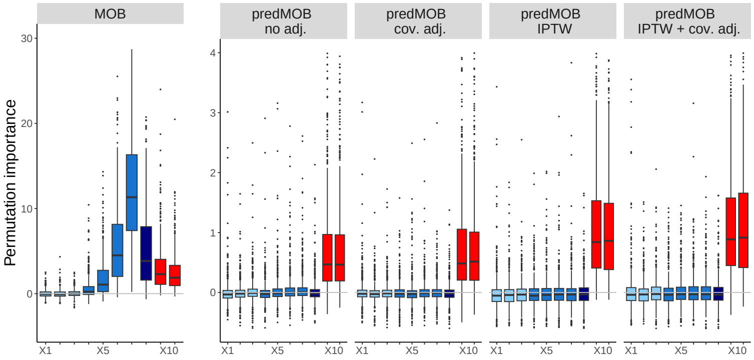

Fig. S.7: **Scenario I2**: Higher order predictive pattern with three-way interaction of X_3, X_9, X_{10} and treatment and high correlation (0.7) between variables X_7, X_9, X_{10} .

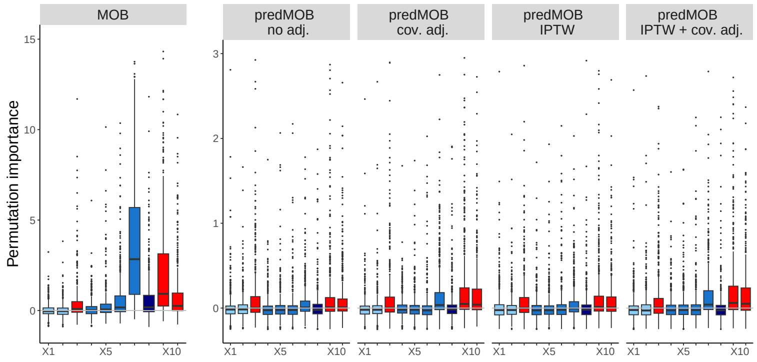

Fig. S.8: **Scenario I3**: Higher order predictive pattern with three-way interaction of X_3, X_9, X_{10} and treatment and high correlation (0.7) between variables X_7, X_9, X_{10}, but variable X_{10} is an unobserved confounder.

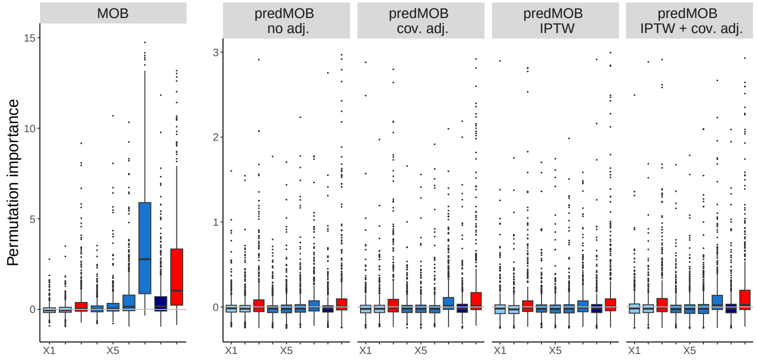

Fig. S.9: **Scenario J2**: Higher dimension with nuisance variables $V_1, \ldots, V_{100}$, $V_{1:5} \sim \text{Bin}(1, 0.5), V_{5:20} \sim \text{Bin}(1, 0.25), V_{80:100} \sim \text{Bin}(1, 0.05)$ added to scenario C

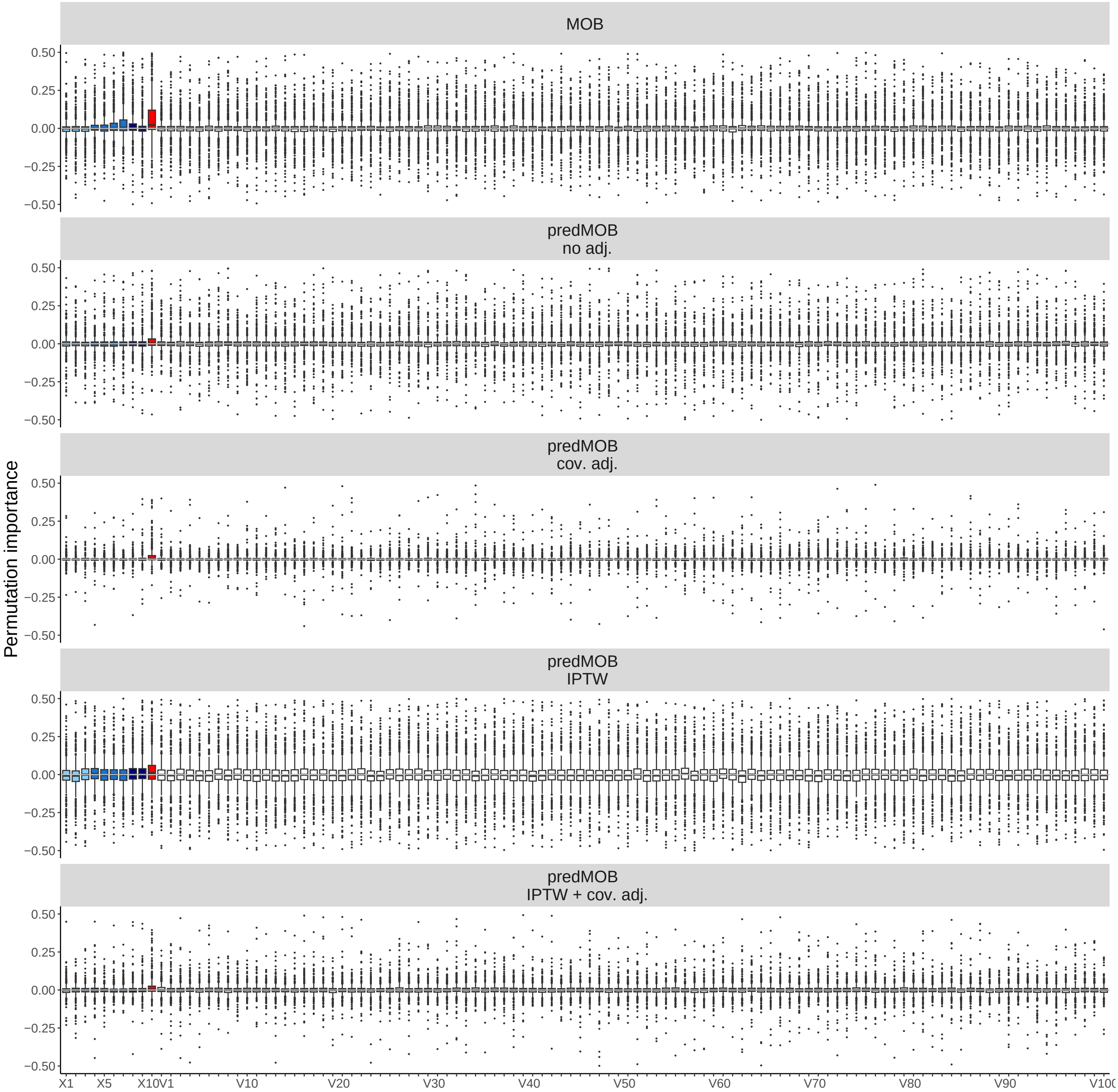

S 2 Additional simulations depicting the need for covariate adjustment

In the simulations presented in the main part of the manuscript (Section 3) the true underlying model for treatment allocation was a linear combination of the observed variables and thus the propensity score model could easily fit the underlying relationships. In this section we present scenarios with a more complex structure such that the linear propensity score model does not adequately fit the underlying structure, thereby leading to false positive findings.

The simulation settings are as described in the simulation section of the manuscript. The binary treatment variable $T \sim B(1, p)$ depends on biomarkers X_6 and X_7 via the following cases, which can also be expressed as a non-linear form:

$$\mathbb{P}(T = 1) = \begin{cases} B(1, 0.5) & \text{if } X_7 = -1 \\ B(1, 0.1) & \text{if } X_7 = 1, X_6 = -1 \\ B(1, 0.9) & \text{if } X_7 = 1, X_6 = 1 \end{cases}$$

The binary distributed outcome variable $Y \sim B(1, \mu)$ with expectation μ:

- **Scenario K**: $\mu = 0.25 \cdot T + 0.8 \cdot X_6 + X_7$
- **Scenario L**: $\mu =$ base formula $+ 0.5 \cdot X_3 \cdot T$
- **Scenario M**: $\mu =$ base formula $+ 0.5 \cdot X_7 \cdot T$

with base formula $= 0.25 \cdot T + 0.1X_4 + 0.15X_5 + 0.2X_6 + 0.25X_7 + 0.2X_8 + 0.1X_9 + 0.15X_{10}$ as in the main simulations (Section 3).

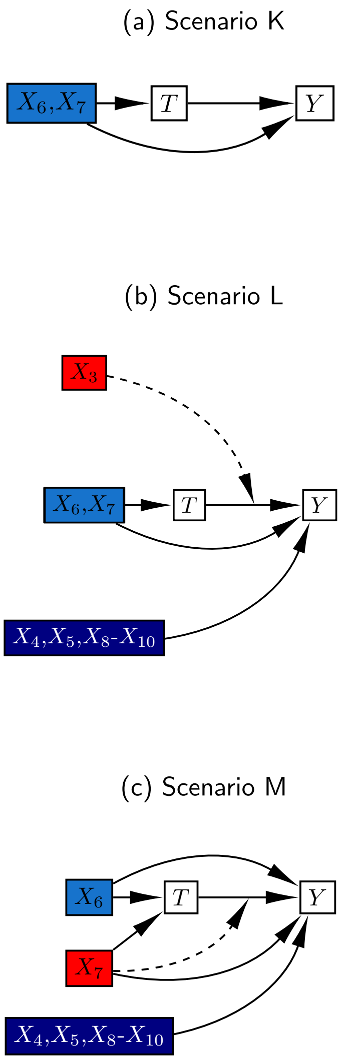

Fig. S.10: Graphical illustration of the additional simulation scenarios K-M. As in the main simulations effect-modification is marked as dashed arrows on the edge representing the treatment effect. Instrumental variables are shown in light blue, true confounders in medium blue and factors only associated with outcome in dark blue; predictive factors are highlighted in red.

Fig. S.11: **Scenario K**: no predictive variables.

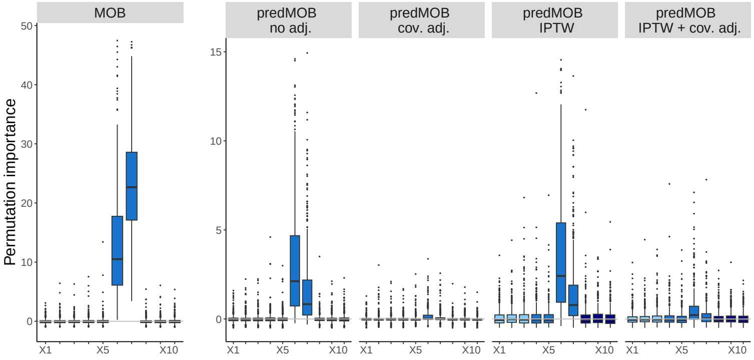

Fig. S.12: **Scenario L**: only X_3 has a predictive effect.

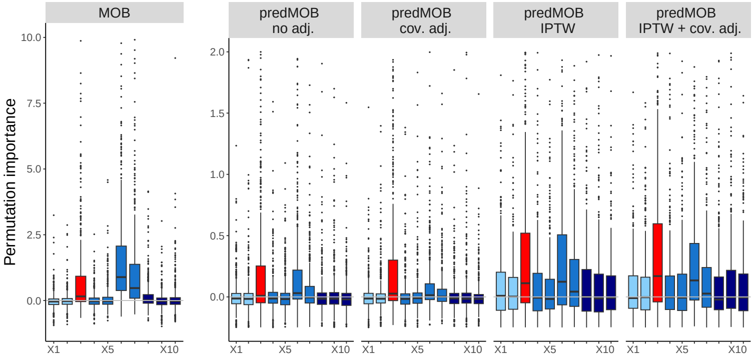

Figures S.11 - S.13 show the results of these simulation scenarios in terms of the permutation importance (note: y-axis has been slightly cropped, thereby excluding a few outliers, for better visual presentation). Furthermore, rank-based presentations of these scenarios are depicted in Supplement Section S 6.1. We observe in Scenario K that without covariate adjustment variable X_6 and X_7 are incorrectly identified by predMOB as predictive factors based on the relatively high permutation importance

Fig. S.13: **Scenario M**: only X_7 has a predictive effect.

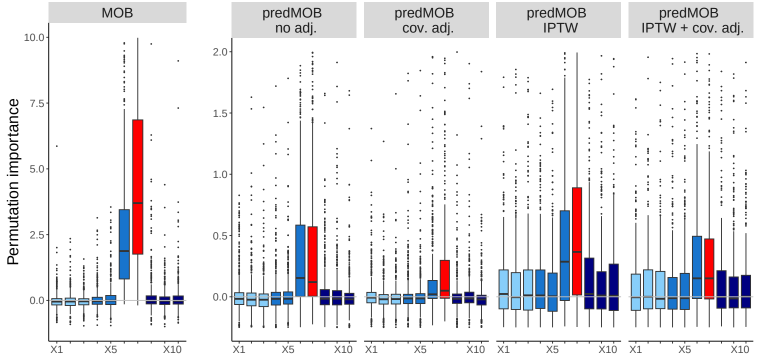

values and consistent ranks 1 and 2. In Scenario L and M the respective predictive factors X_3 and X_7 are correctly identified in terms of relatively high permutation importance and often top rank, but also X_6 shows an increased permutation importance and top rank even though it is not predictive. When using predMOB without covariate adjustment the influence of X_6 and X_7 on treatment assignment can lead to a false positive finding for these variables as predictive factors. When using IPTW we also have increased chances of false positives due to the fact that the true non-linear association in the treatment assignment is not well represented by the linear propensity score model that was fitted. In these scenarios predMOB with covariate adjustment shows the most promising results, followed by its combination with IPTW.

S 3 Additional simulations depicting the need for IPTW

We show additional scenarios for cases where the true model underlying the treatment assignment is linear and the outcome model is not. In these scenarios the propensity score model can be used as a suitable approximation for the treatment assignment, but the linear model fitted for the outcome is not correct as it does not take into account the interaction.

Specifically we model the binary treatment variable T as in the main section of the simulations (Section 3) with $T \sim B(1, p)$; depending on biomarkers $X_1, \ldots, X_7$ via the logistic regression model

$\text{logit}(p) = \beta_0 + \log(1.1)X_1 - \log(1.2)X_2 + \log(1.3)X_3 - \log(1.1)X_4 + \log(1.2)X_5 - \log(1.3)X_6 + \log(1.4)X_7$,

with β_0 being chosen so $p = 0.5$,

The binary distributed outcome variable $Y \sim B(1, \pi)$ with $logit(\pi) = \mu$:

- **Scenario N**:

$$\mu_{scenarioN} = 0.5 \cdot T + \begin{cases} 0.75 & \text{if } X_7 = -1 \\ -0.8 & \text{if } X_7 = 1, X_6 = -1 \\ +1.5 & \text{if } X_7 = 1, X_6 = 1 \end{cases}$$

- **Scenario P**: $\mu_{scenarioP} = \mu_{scenarioN} + 1.5 \cdot X_{10} \cdot T$

Figures S.15 - S.16 show the results of these simulation scenarios in terms of the permutation importance. See Appendix Section S 6.1 for rank-based results.

In scenarios N and P, where there is a non-linear relationship between variables X_6, X_7 and the outcome, we observe that predMOB with no adjustment and covariate adjustment (which assumes a linear relationship) indicate false positive findings in terms of possible predictive effects for X_6, X_7 represented by relatively high permutation importance values and consistently top ranks. The predMOB methods with IPTW have an advantage in these scenarios as the true propensity score model is in

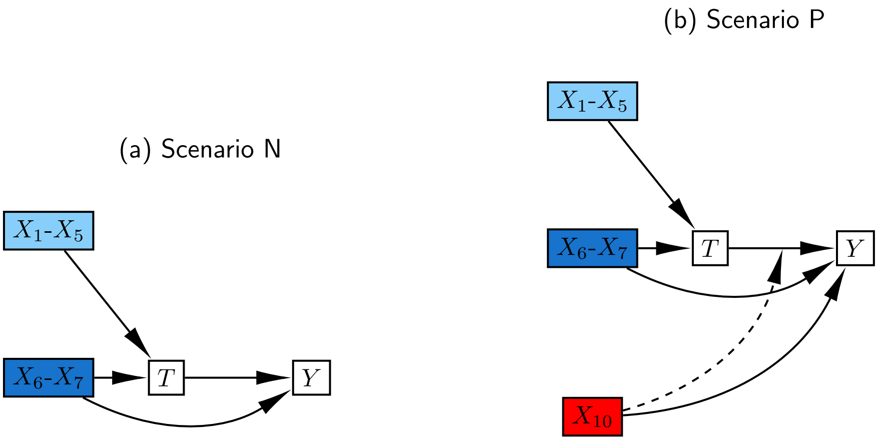

Fig. S.14: Graphical illustration of the additional simulation scenarios N and P. As in the main simulations effect-modification is marked as dashed arrows on the edge representing the treatment effect. Instrumental variables are shown in light blue, true confounders in medium blue and factors only associated with outcome in dark blue; predictive factors are highlighted in red.

Fig. S.15: **Scenario N**: no predictive variables.

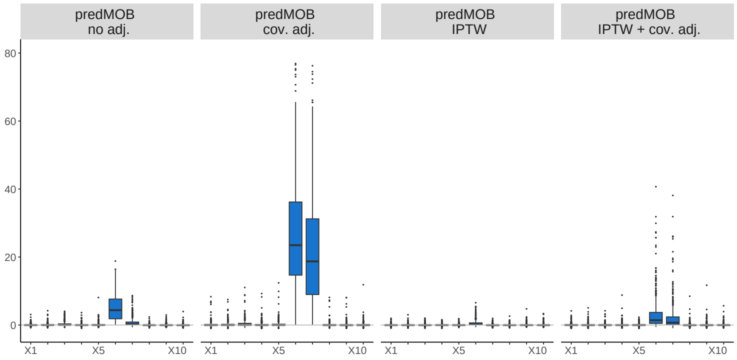

fact linear and can thus be appropriately modeled with a linear fit leading to a reduced risk of false positives. In scenario P variable X_{10} has a predictive effect and can be correctly identified by all predMOB approaches in terms of relatively high permutation importance and top rank, albeit X_6 and X_7 are still falsely identified as potential variables with predictive effects.

Fig. S.16: **Scenario P**: only X_{10} has a predictive effect.

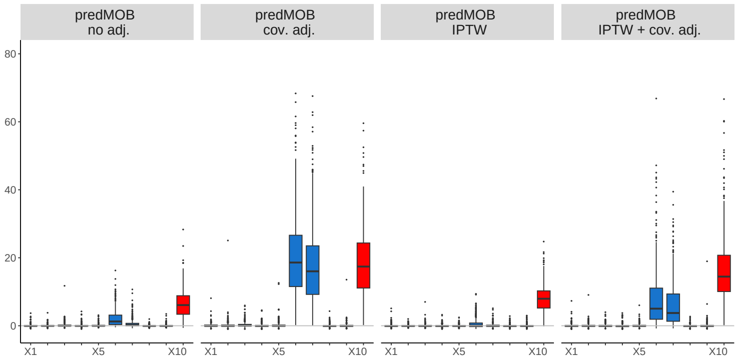

S 4 Additional outputs for application example of GBSG2 trial

Descriptive statistics for the two GBSG2 trial subpopulations by hormonal therapy yes/no are tabulated in Supplementary Table S.1.

Figure S.17 displays the absolute mean differences in the covariates between the two treatment arms in the non-randomized subpopulation. The red dots reveal that the covariates with the largest imbalance are menopausal status, estrogen receptor status and tumor grade. After using IPTW, the mean differences in the weighted population (marked in blue) are close to zero.

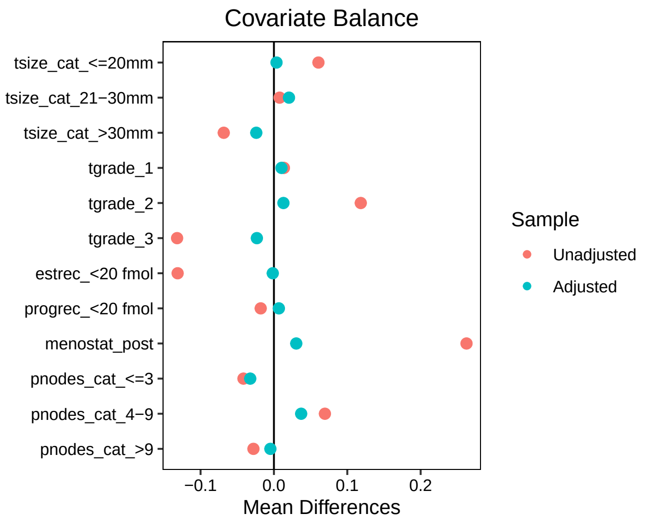

Fig. S.17: Comparison of difference in means between patients of the non-randomized population treated with or without tamoxifen for potential confounder variables. Red dots represent the unadjusted data while blue dots display the differences after IPTW. The differences in menopausal status, estrogen receptor status and tumor grade can be balanced out by IPTW.

Table S.1: Descriptive statistics for GBSG2 data by subpopulation and hormonal therapy

	Randomized			Non-Randomized		
	**no Tamoxifen (N=189)**	**Tamoxifen (N=184)**	**All (N=373)**	**no Tamoxifen (N=176)**	**Tamoxifen (N=71)**	**All (N=247)**
**Age [years]**						
Mean (SD)	54.6 (9.76)	56.0 (9.34)	55.3 (9.57)	51.2 (9.85)	57.9 (9.56)	53.2 (10.2)
Median [Min, Max]	55.0 [25.0, 80.0]	58.0 [33.0, 72.0]	57.0 [25.0, 80.0]	50.0 [21.0, 76.0]	58.0 [32.0, 80.0]	52.0 [21.0, 80.0]
**Chemotherapy**						
3xCMF	93 (49.2%)	93 (50.5%)	186 (49.9%)	72 (40.9%)	42 (59.2%)	114 (46.2%)
6xCMF	96 (50.8%)	91 (49.5%)	187 (50.1%)	104 (59.1%)	29 (40.8%)	133 (53.8%)
**Number positive lymph nodes**						
≤3	115 (60.8%)	95 (51.6%)	210 (56.3%)	92 (52.3%)	37 (52.1%)	129 (52.2%)
4-9	56 (29.6%)	62 (33.7%)	118 (31.6%)	50 (28.4%)	23 (32.4%)	73 (29.6%)
>9	16 (8.5%)	27 (14.7%)	43 (11.5%)	32 (18.2%)	10 (14.1%)	42 (17.0%)
Missing	2 (1.1%)	0 (0%)	2 (0.5%)	2 (1.1%)	1 (1.4%)	3 (1.2%)
**Menopausal Status**						
pre	55 (29.1%)	45 (24.5%)	100 (26.8%)	89 (50.6%)	17 (23.9%)	106 (42.9%)
post	134 (70.9%)	139 (75.5%)	273 (73.2%)	87 (49.4%)	54 (76.1%)	141 (57.1%)
**Tumor Size [mm]**						

≤20 mm	55 (29.1%)	48 (26.1%)	103 (27.6%)	39 (22.2%)	20 (28.2%)	59 (23.9%)
21-30 mm	74 (39.2%)	79 (42.9%)	153 (41.0%)	76 (43.2%)	30 (42.3%)	106 (42.9%)
>30 mm	59 (31.2%)	56 (30.4%)	115 (30.8%)	61 (34.7%)	21 (29.6%)	82 (33.2%)
Missing	1 (0.5%)	1 (0.5%)	2 (0.5%)	0 (0%)	0 (0%)	0 (0%)
**Tumor grade**						
1	22 (11.6%)	25 (13.6%)	47 (12.6%)	21 (11.9%)	9 (12.7%)	30 (12.1%)
2	118 (62.4%)	118 (64.1%)	236 (63.3%)	102 (58.0%)	51 (71.8%)	153 (61.9%)
3	43 (22.8%)	41 (22.3%)	84 (22.5%)	51 (29.0%)	11 (15.5%)	62 (25.1%)
Missing	6 (3.2%)	0 (0%)	6 (1.6%)	2 (1.1%)	0 (0%)	2 (0.8%)
**Estrogen receptors [fmol/l]**						
≥20 fmol	116 (61.4%)	109 (59.2%)	225 (60.3%)	104 (59.1%)	53 (74.6%)	157 (63.6%)
<20 fmol	68 (36.0%)	71 (38.6%)	139 (37.3%)	69 (39.2%)	17 (23.9%)	86 (34.8%)
Missing	5 (2.6%)	4 (2.2%)	9 (2.4%)	3 (1.7%)	1 (1.4%)	4 (1.6%)
**Progesterone receptors [fmol/l]**						
≥20 fmol	107 (56.6%)	102 (55.4%)	209 (56.0%)	106 (60.2%)	47 (66.2%)	153 (61.9%)
<20 fmol	75 (39.7%)	79 (42.9%)	154 (41.3%)	66 (37.5%)	23 (32.4%)	89 (36.0%)
Missing	7 (3.7%)	3 (1.6%)	10 (2.7%)	4 (2.3%)	1 (1.4%)	5 (2.0%)
**RFS**						
≥ 2 years	137 (72.5%)	130 (70.7%)	267 (71.6%)	119 (67.6%)	57 (80.3%)	176 (71.3%)
< 2 years	48 (25.4%)	46 (25.0%)	94 (25.2%)	48 (27.3%)	13 (18.3%)	61 (24.7%)

Censored	4 (2.1%)	8 (4.3%)	12 (3.2%)	9 (5.1%)	1 (1.4%)	10 (4.0%)

Variable	estimate	lower	upper	pValue
Tamoxifen treatment	-0.85	-2.47	0.76	0.3
Tumor size (21-30 mm)	0.13	-0.83	1.10	0.787
Tumor size ($\geq$ 30 mm)	0.41	-0.58	1.40	0.415
Progesterone receptor status ($\leq$20 fmol)	0.85	-0.18	1.87	0.104
Estrogen receptor status ($\leq$20 fmol)	-0.51	-1.60	0.58	0.357
Post menopausal status	-0.69	-1.52	0.13	0.1
Number positive lymph nodes (4-9)	1.04	0.24	1.83	0.011
Number positive lymph nodes ($\geq$9)	1.32	0.11	2.53	0.033
Tumor grade 2	2.19	0.13	4.24	0.037
Tumor grade 3	2.67	0.55	4.79	0.014
Tamoxifen treatment * Tumor size (21-30 mm)	-0.03	-1.50	1.44	0.966
Tamoxifen treatment * Tumor size ($\geq$30 mm)	-0.20	-1.72	1.32	0.798
Tamoxifen treatment * Progesterone receptor status ($\leq$20 fmol)	0.67	-0.79	2.14	0.367
Tamoxifen treatment * Estrogen receptor status ($\leq$20 fmol)	0.41	-1.12	1.94	0.599
Tamoxifen treatment * Post menopausal status	0.39	-0.77	1.55	0.511
Tamoxifen treatment * Number positive lymph nodes (4-9)	-0.08	-1.25	1.09	0.896
Tamoxifen treatment * Number positive lymph nodes ($\geq$9)	0.02	-1.62	1.66	0.982

Table S.2: Results for randomized cohort applying logistic regression with all possible treatment interactions. Note: treatment interaction with tumor grade was excluded due to poor model fit. Abbreviations: lower/upper 95% confidence limits.

Variable	estimate	lower	upper	pValue
Tamoxifen treatment	-0.01	-3.28	3.26	0.995
Tumor size (21-30 mm)	1.43	0.30	2.55	0.013
Tumor size ($\geq$ 30 mm)	1.24	0.07	2.42	0.039
Tumor grade 2	-0.19	-1.37	1.00	0.756
Tumor grade 3	0.10	-1.41	1.61	0.898
Progesterone receptor status ($\leq$20 fmol)	0.63	-0.23	1.50	0.149
Estrogen receptor status ($\leq$20 fmol)	0.61	-0.26	1.49	0.166
Post menopausal status	0.47	-0.40	1.33	0.287
Number positive lymph nodes (4-9)	0.07	-0.81	0.95	0.877
Number positive lymph nodes ($\geq$9)	1.53	0.50	2.55	0.004
Tamoxifen treatment * Tumor size (21-30 mm)	-1.43	-4.05	1.19	0.284
Tamoxifen treatment * Tumor size ($\geq$30 mm)	-0.78	-3.82	2.26	0.614
Tamoxifen treatment * Tumor grade 2	-1.05	-4.30	2.19	0.523
Tamoxifen treatment * Tumor grade 3	0.13	-2.75	3.02	0.928
Tamoxifen treatment * Progesterone receptor status ($\leq$20 fmol)	2.83	0.23	5.43	0.033
Tamoxifen treatment * Estrogen receptor status ($\leq$20 fmol)	-1.29	-3.51	0.93	0.254
Tamoxifen treatment * Post menopausal status	-0.21	-2.25	1.84	0.843
Tamoxifen treatment * Number positive lymph nodes (4-9)	1.21	-0.87	3.29	0.253
Tamoxifen treatment * Number positive lymph nodes ($\geq$9)	-1.39	-3.90	1.12	0.276

Table S.3: Results for non-randomized cohort applying logistic regression with all possible treatment interactions. Abbreviations: lower/upper 95% confidence limits.

S 5 Additional outputs for application example of AMLSG 16-10 trial

Variable	estimate	lower	upper	pValue
AMLSG 16-10 (active treatment)	-0.80	-2.63	1.02	0.389
Age	-0.02	-0.04	-0.00	0.028
Female sex	0.37	-0.07	0.80	0.096
NPM1 mutated	1.34	0.89	1.79	<.001
WBC (log10)	-0.57	-0.98	-0.16	0.007
BM blasts	0.11	-1.04	1.26	0.847
FLT3-ITD high	-0.46	-1.02	0.11	0.113
AMLSG 16-10 (active trt) * Age	0.01	-0.02	0.04	0.426
AMLSG 16-10 (active trt) * female sex	-0.65	-1.29	-0.01	0.047
AMLSG 16-10 (active trt) * NPM1 mutated	0.04	-0.62	0.69	0.91
AMLSG 16-10 (active trt) * WBC (log10)	0.38	-0.20	0.96	0.196
AMLSG 16-10 (active trt) * BM blasts	0.42	-1.19	2.04	0.605
AMLSG 16-10 (active trt) * FLT3-ITD high	0.39	-0.35	1.14	0.298

Table S.4: Results for response to induction therapy (CR/CRi) from logistic regression model with all treatment interactions. Abbreviations: BM, bone marrow; CR, complete remission; CRi, CR with incomplete hematologic recovery; ITD, internal tandem duplication; OR, odds ratio; WBC, white blood cells; lower/upper 95% confidence limits

S 6 Rank-based presentation of results

S 6.1 Simulation results

In this section we present an alternative depiction of the simulation results where each variable is ranked by highest to smallest variable importance score across simulation runs for the different methods in various settings. The plots show the distribution of the ranks across the simulation runs (percentages of observed ranks presented by the size of dots as a measure of ranking stability) and the region between the 2.5% and the 97.5% percentile presented by the connecting line. Ties are given maximum rank. Note: ties occur almost exclusively for variable importance values equal to 0, meaning that the respective variable was not selected for any split in the tree, and thus has no prognostic/predictive effect.

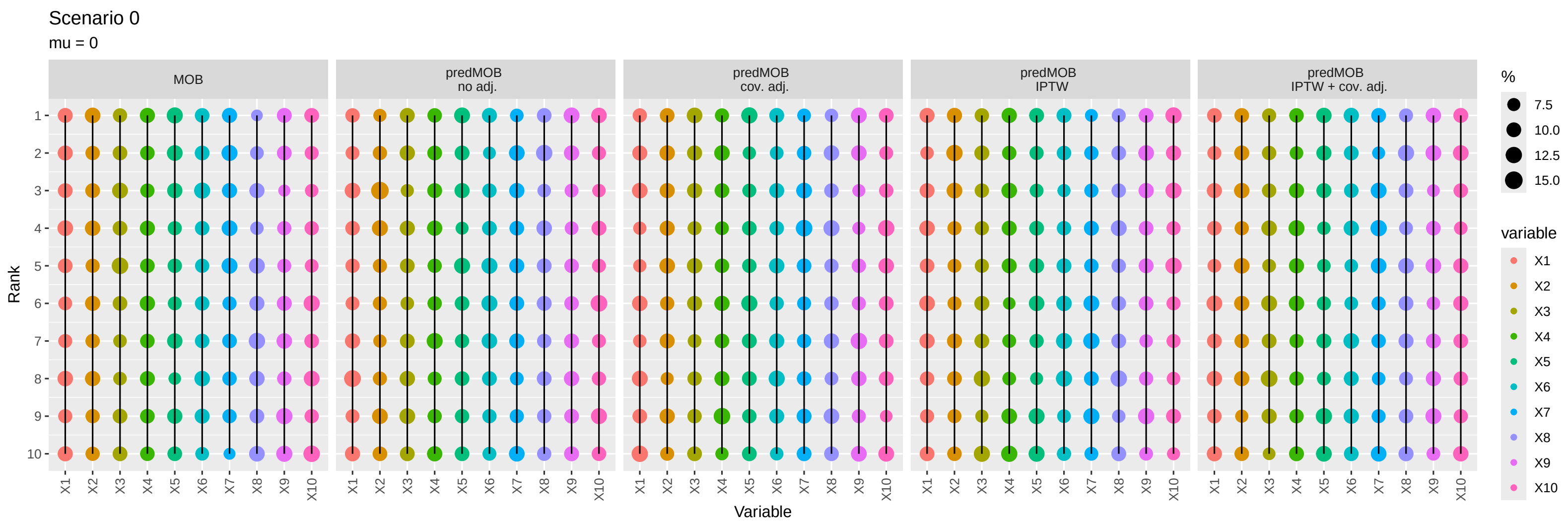

Scenario 0
mu = 0
MOB
predMOB no adj.
predMOB cov. adj.
predMOB IPTW
predMOB IPTW + cov. adj.
Rank
Variable
%
7.5
10.0
12.5
15.0
variable
X1
X2
X3
X4
X5
X6
X7
X8
X9
X10

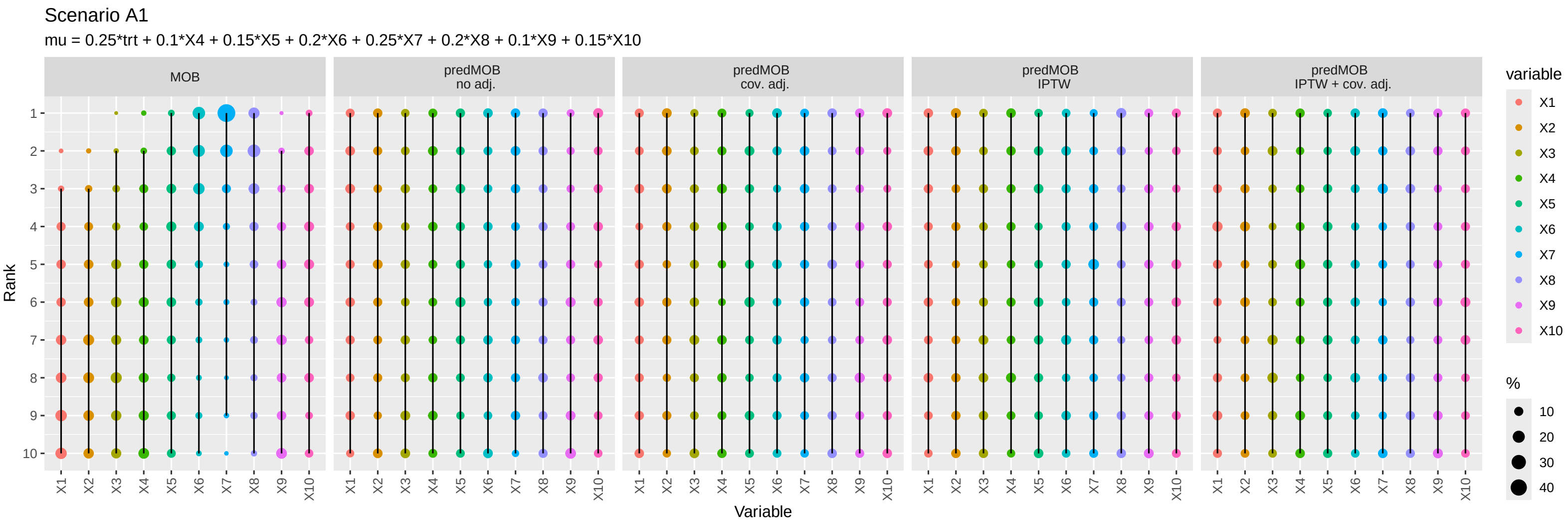
Scenario A1
mu = 0.25*trt + 0.1*X4 + 0.15*X5 + 0.2*X6 + 0.25*X7 + 0.2*X8 + 0.1*X9 + 0.15*X10
MOB
predMOB no adj.
predMOB cov. adj.
predMOB IPTW
predMOB IPTW + cov. adj.
Rank
Variable
variable
X1
X2
X3
X4
X5
X6
X7
X8
X9
X10
%
10
20
30
40

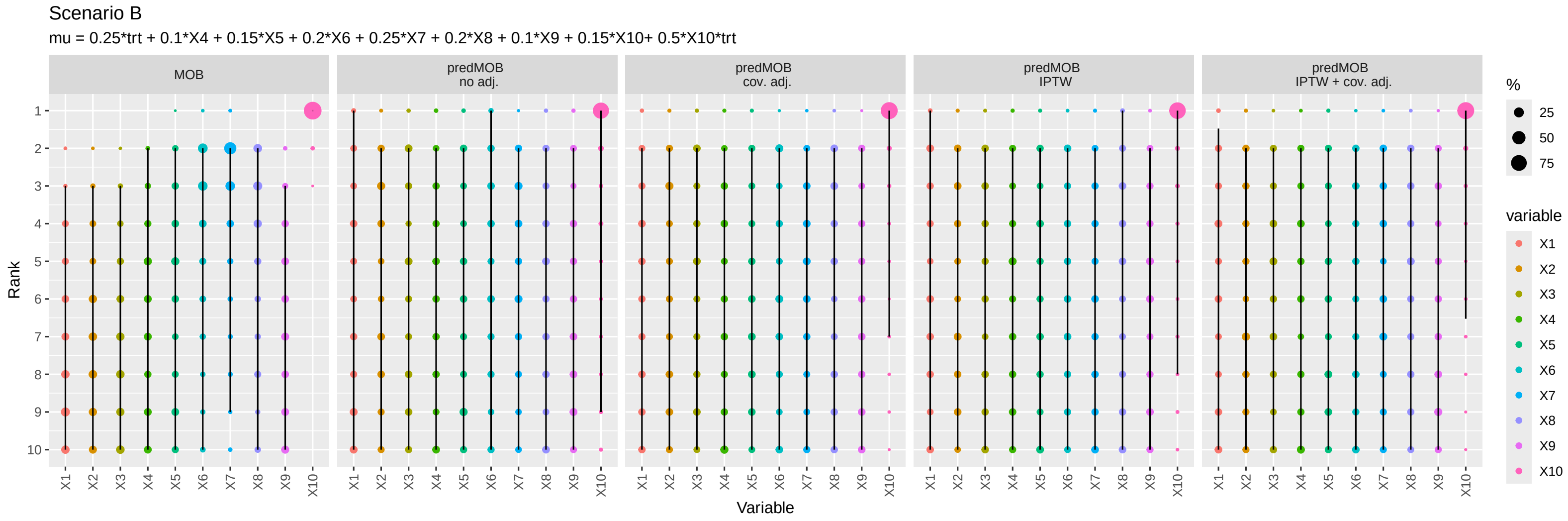

Scenario B
mu = 0.25*trt + 0.1*X4 + 0.15*X5 + 0.2*X6 + 0.25*X7 + 0.2*X8 + 0.1*X9 + 0.15*X10+ 0.5*X10*trt
MOB
predMOB no adj.
predMOB cov. adj.
predMOB IPTW
predMOB IPTW + cov. adj.
Rank
Variable
%
25
50
75
variable
X1
X2
X3
X4
X5
X6
X7
X8
X9
X10

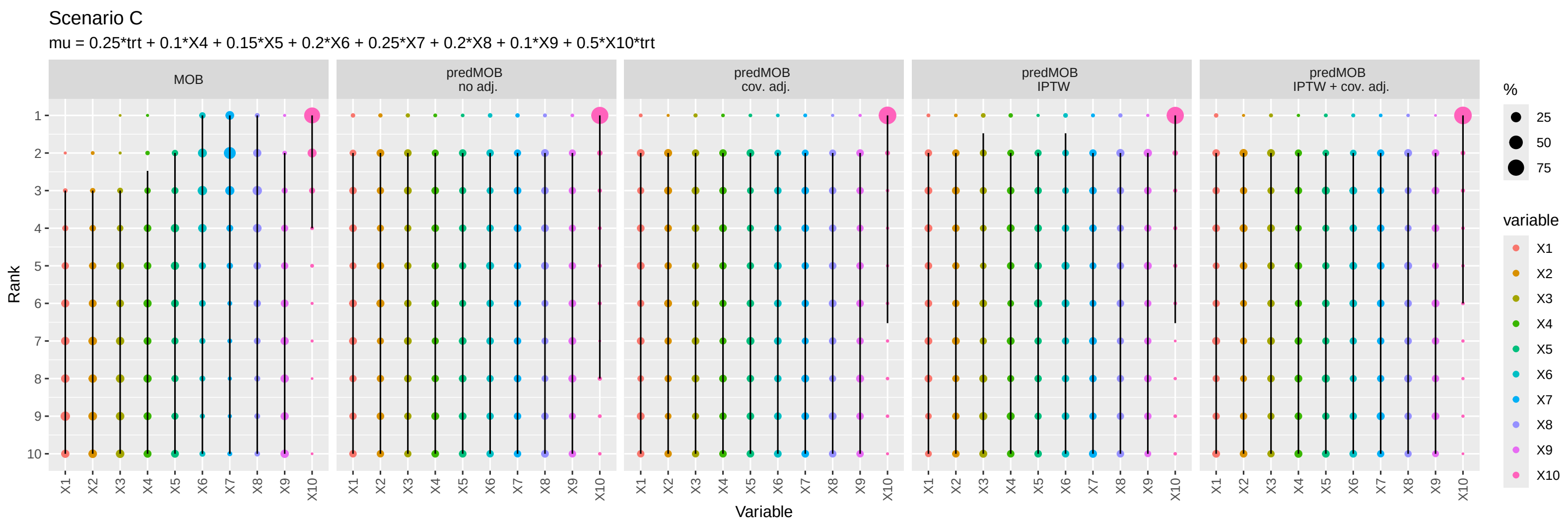

Scenario C
mu = 0.25*trt + 0.1*X4 + 0.15*X5 + 0.2*X6 + 0.25*X7 + 0.2*X8 + 0.1*X9 + 0.5*X10*trt
MOB
predMOB no adj.
predMOB cov. adj.
predMOB IPTW
predMOB IPTW + cov. adj.
Rank
Variable
%
25
50
75
variable
X1
X2
X3
X4
X5
X6
X7
X8
X9
X10

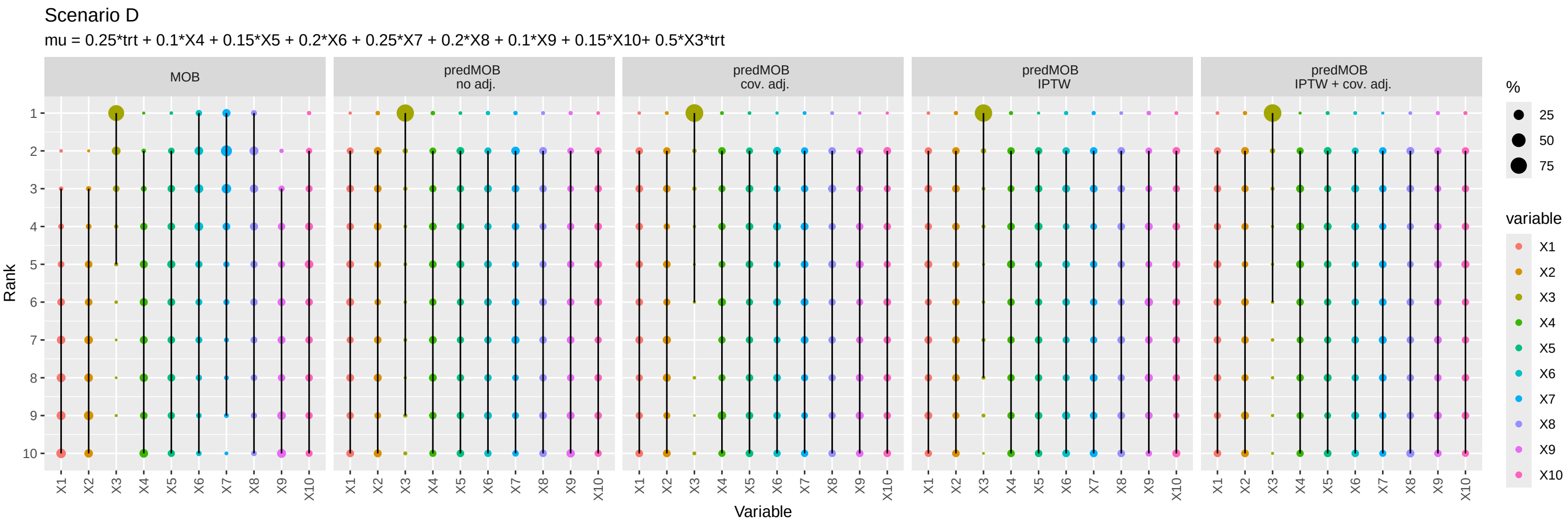
Scenario D
mu = 0.25*trt + 0.1*X4 + 0.15*X5 + 0.2*X6 + 0.25*X7 + 0.2*X8 + 0.1*X9 + 0.15*X10+ 0.5*X3*trt
MOB
predMOB no adj.
predMOB cov. adj.
predMOB IPTW
predMOB IPTW + cov. adj.
Rank
Variable
%
25
50
75
variable
X1
X2
X3
X4
X5
X6
X7
X8
X9
X10

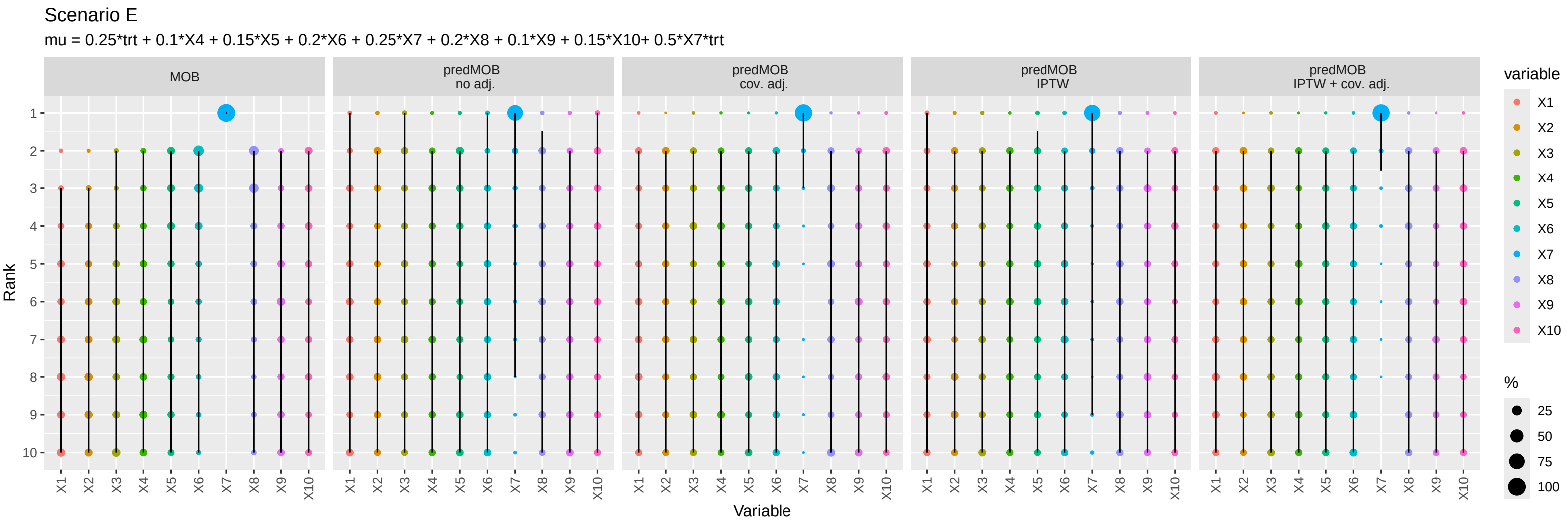
Scenario E
mu = 0.25*trt + 0.1*X4 + 0.15*X5 + 0.2*X6 + 0.25*X7 + 0.2*X8 + 0.1*X9 + 0.15*X10+ 0.5*X7*trt
MOB
predMOB
no adj.
predMOB
cov. adj.
predMOB
IPTW
predMOB
IPTW + cov. adj.
Rank
Variable
variable
X1
X2
X3
X4
X5
X6
X7
X8
X9
X10
%
25
50
75
100

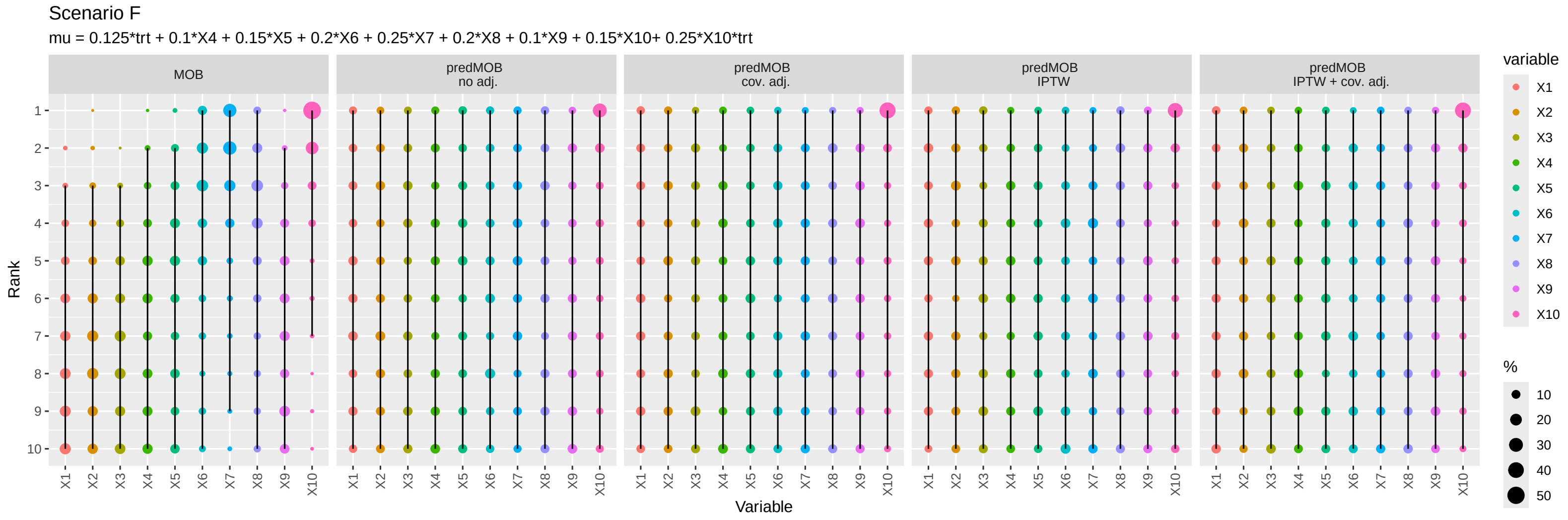
Scenario F
mu = 0.125*trt + 0.1*X4 + 0.15*X5 + 0.2*X6 + 0.25*X7 + 0.2*X8 + 0.1*X9 + 0.15*X10+ 0.25*X10*trt
MOB
predMOB no adj.
predMOB cov. adj.
predMOB IPTW
predMOB IPTW + cov. adj.
Rank
Variable
variable
X1
X2
X3
X4
X5
X6
X7
X8
X9
X10
%
10
20
30
40
50

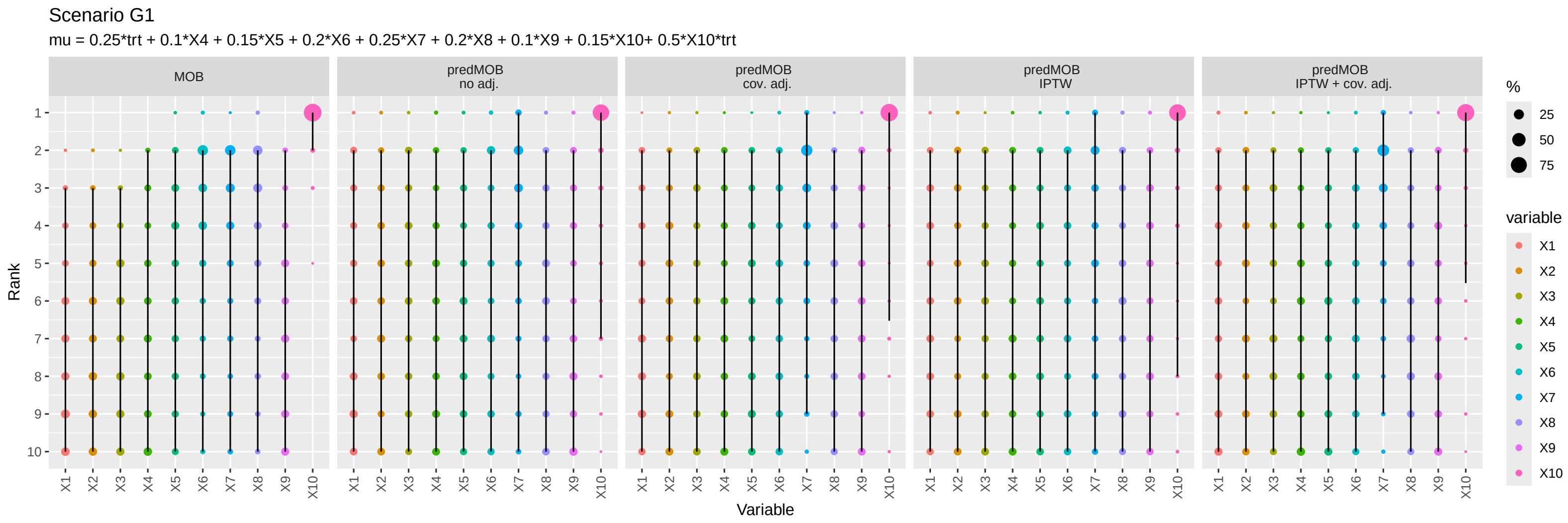
Scenario G1
mu = 0.25*trt + 0.1*X4 + 0.15*X5 + 0.2*X6 + 0.25*X7 + 0.2*X8 + 0.1*X9 + 0.15*X10+ 0.5*X10*trt
MOB
predMOB
no adj.
predMOB
cov. adj.
predMOB
IPTW
predMOB
IPTW + cov. adj.
Rank
Variable
%
25
50
75
variable
X1
X2
X3
X4
X5
X6
X7
X8
X9
X10

Scenario G2

mu = 0.25*trt + 0.1*X4 + 0.15*X5 + 0.2*X6 + 0.25*X7 + 0.2*X8 + 0.1*X9 + 0.15*X10+ 0.5*X10*trt

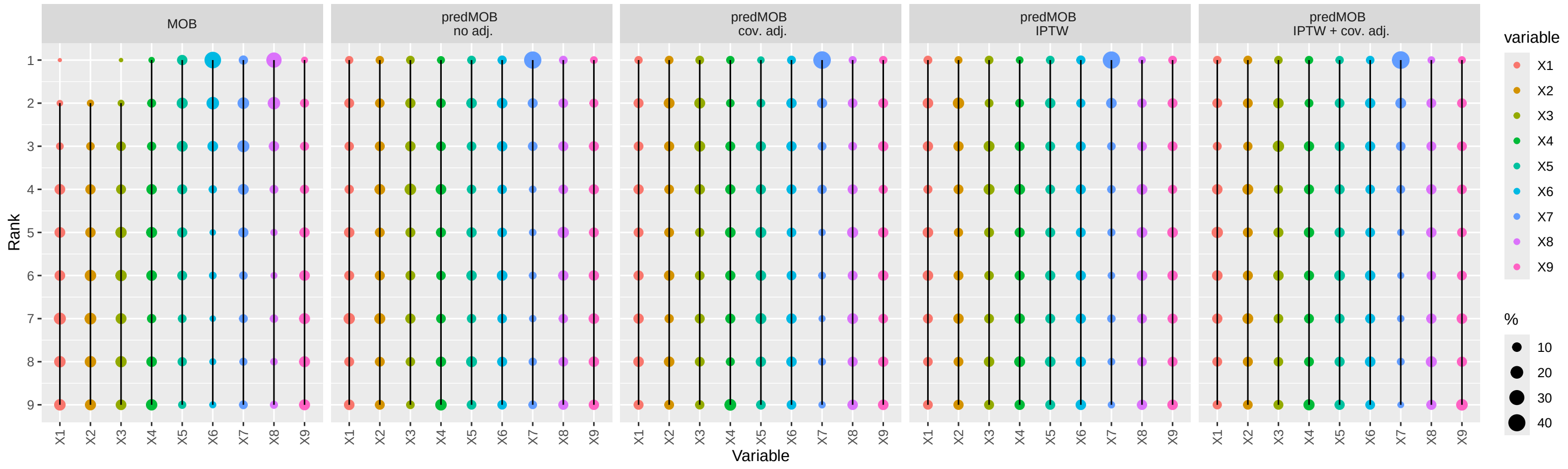

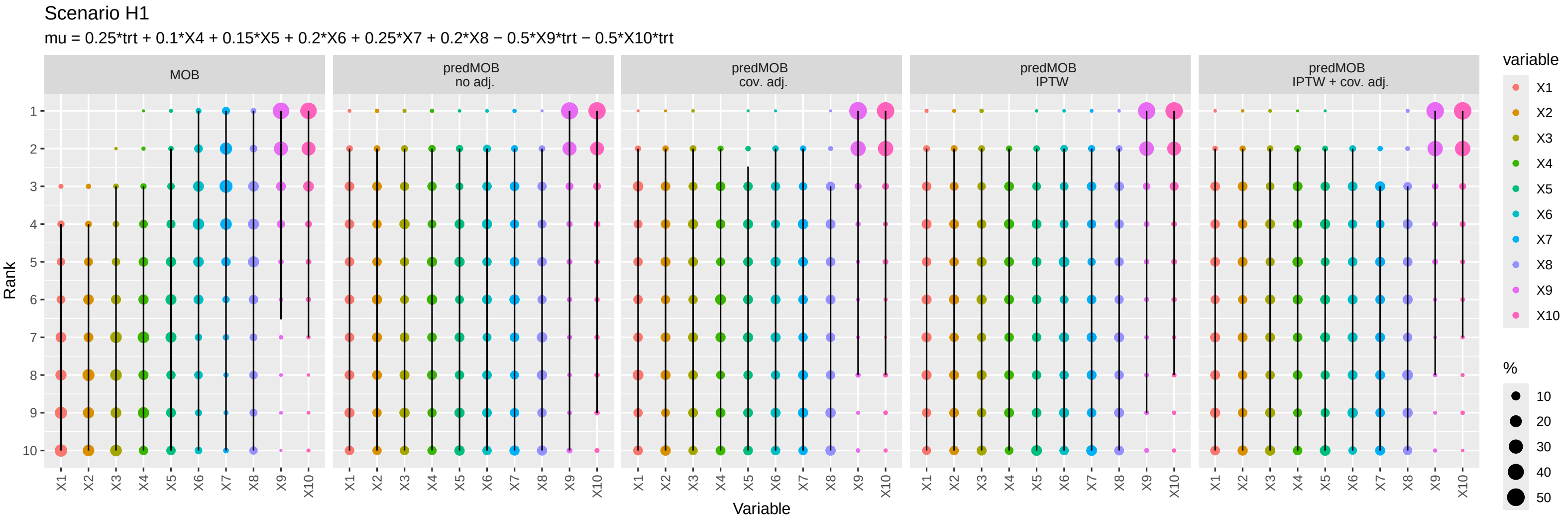
Scenario H1
mu = 0.25*trt + 0.1*X4 + 0.15*X5 + 0.2*X6 + 0.25*X7 + 0.2*X8 − 0.5*X9*trt − 0.5*X10*trt
MOB
predMOB
no adj.
predMOB
cov. adj.
predMOB
IPTW
predMOB
IPTW + cov. adj.
Rank
Variable
variable
X1
X2
X3
X4
X5
X6
X7
X8
X9
X10
%
10
20
30
40
50

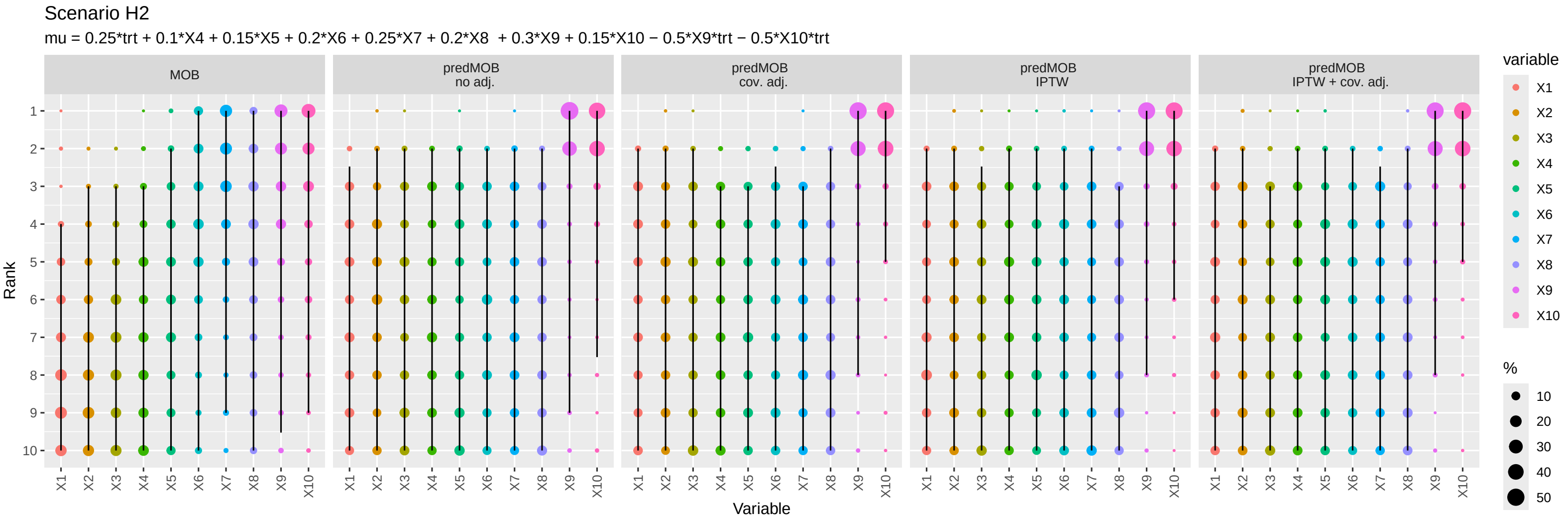
Scenario H2
mu = 0.25*trt + 0.1*X4 + 0.15*X5 + 0.2*X6 + 0.25*X7 + 0.2*X8 + 0.3*X9 + 0.15*X10 − 0.5*X9*trt − 0.5*X10*trt
MOB
predMOB no adj.
predMOB cov. adj.
predMOB IPTW
predMOB IPTW + cov. adj.
Rank
Variable
variable
X1 X2 X3 X4 X5 X6 X7 X8 X9 X10
%
10 20 30 40 50

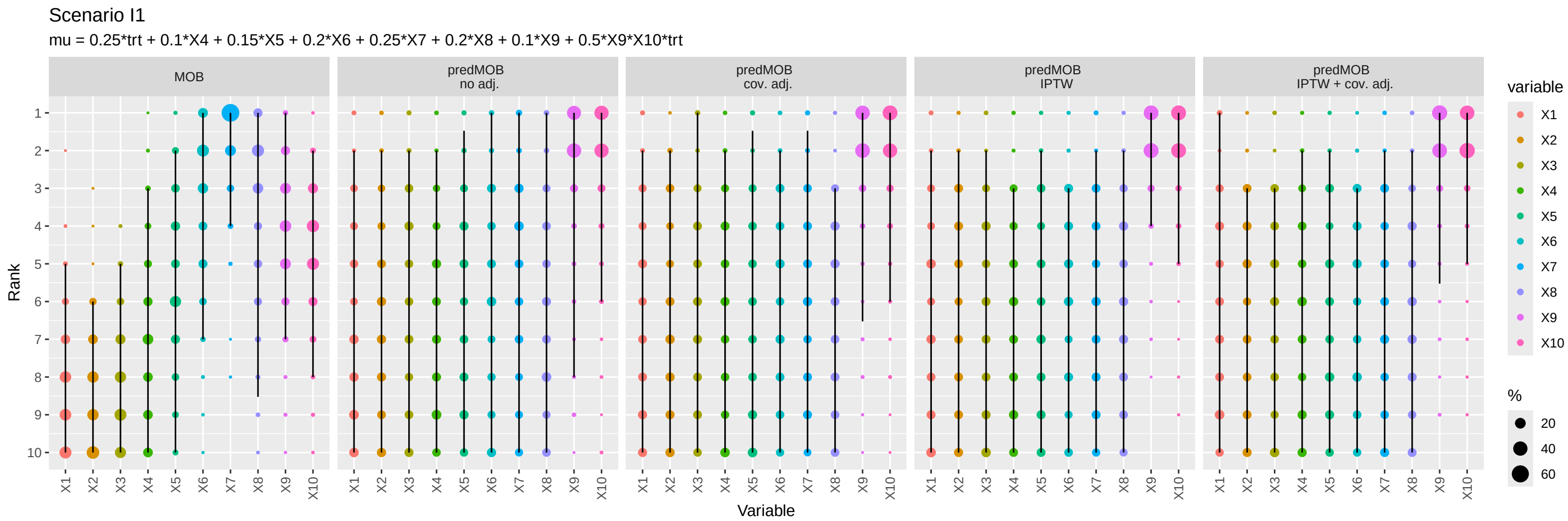

Scenario I1
mu = 0.25*trt + 0.1*X4 + 0.15*X5 + 0.2*X6 + 0.25*X7 + 0.2*X8 + 0.1*X9 + 0.5*X9*X10*trt
MOB
predMOB no adj.
predMOB cov. adj.
predMOB IPTW
predMOB IPTW + cov. adj.
Rank
Variable
variable
X1
X2
X3
X4
X5
X6
X7
X8
X9
X10
%
20
40
60

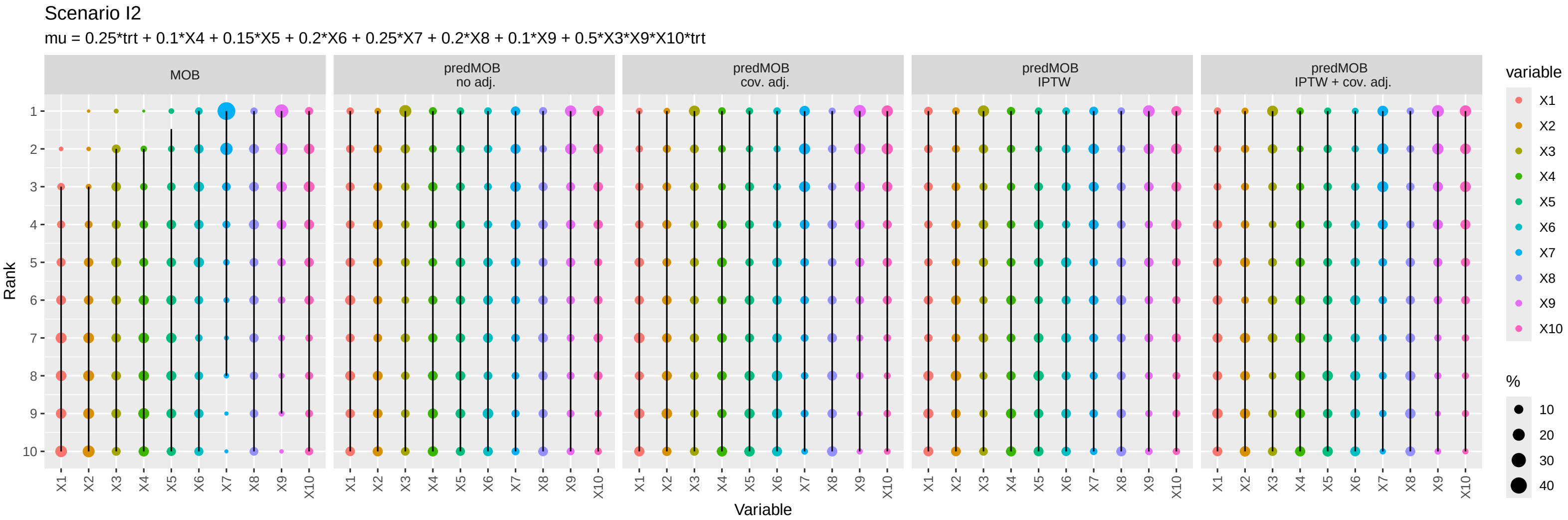

Scenario I2
mu = 0.25*trt + 0.1*X4 + 0.15*X5 + 0.2*X6 + 0.25*X7 + 0.2*X8 + 0.1*X9 + 0.5*X3*X9*X10*trt
MOB
predMOB no adj.
predMOB cov. adj.
predMOB IPTW
predMOB IPTW + cov. adj.
Rank
Variable
variable
X1
X2
X3
X4
X5
X6
X7
X8
X9
X10
%
10
20
30
40

Scenario I3

mu = 0.25*trt + 0.1*X4 + 0.15*X5 + 0.2*X6 + 0.25*X7 + 0.2*X8 + 0.1*X9 + 0.5*X3*X9*X10*trt

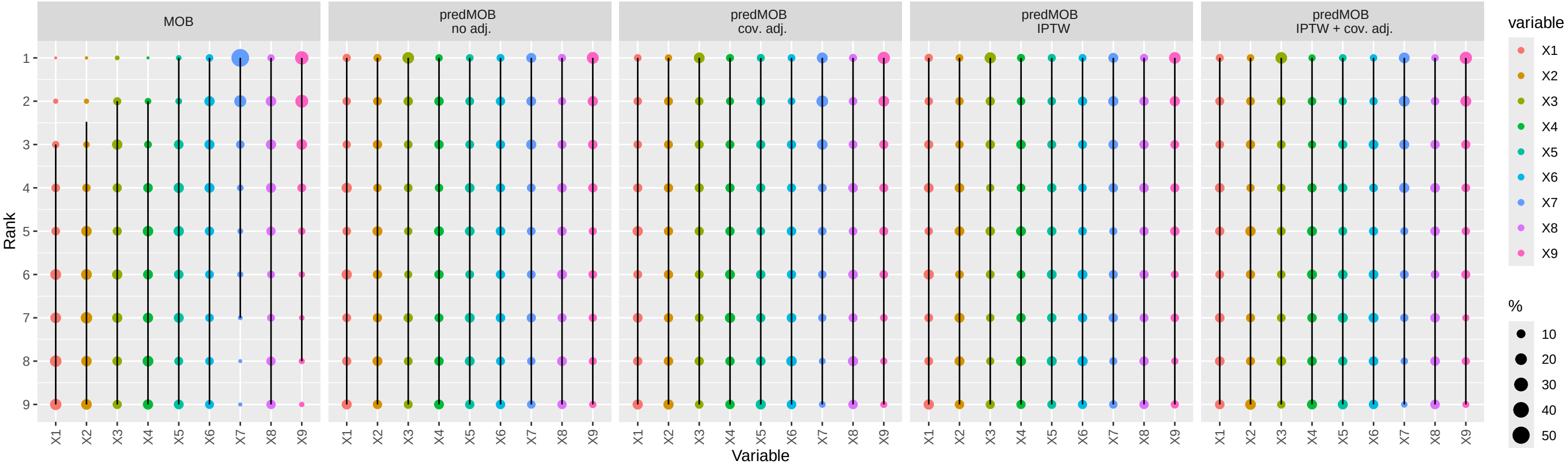

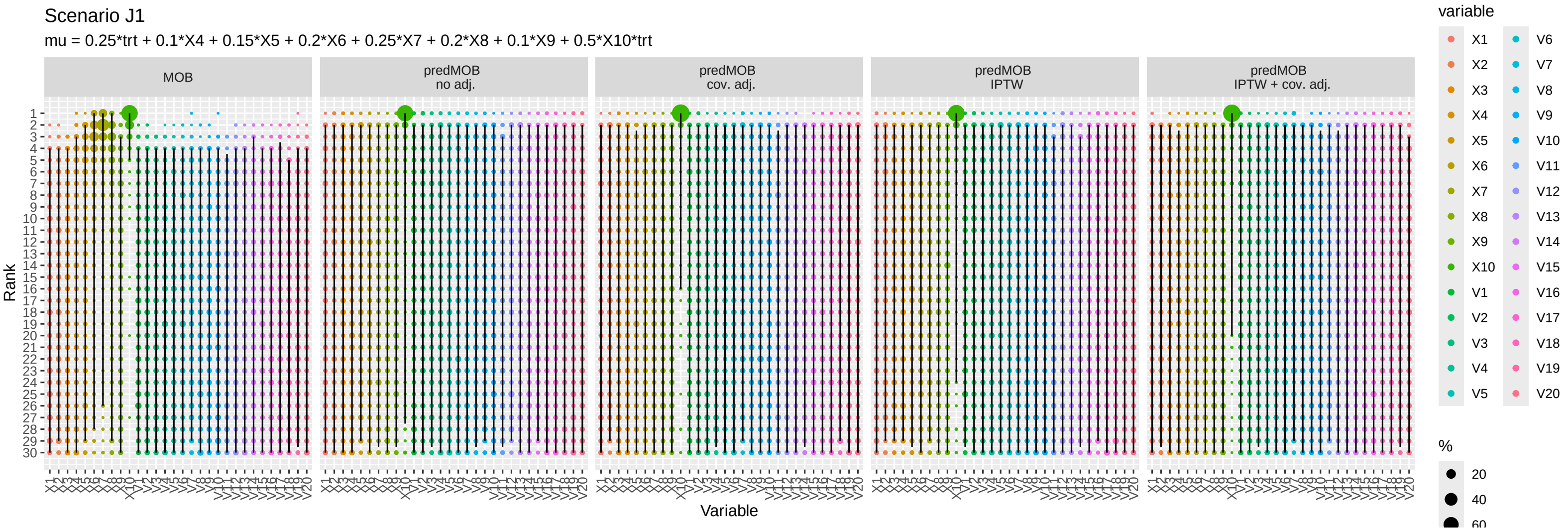

Scenario J1
mu = 0.25*trt + 0.1*X4 + 0.15*X5 + 0.2*X6 + 0.25*X7 + 0.2*X8 + 0.1*X9 + 0.5*X10*trt
MOB
predMOB no adj.
predMOB cov. adj.
predMOB IPTW
predMOB IPTW + cov. adj.
Rank
Variable
variable
%
20
40

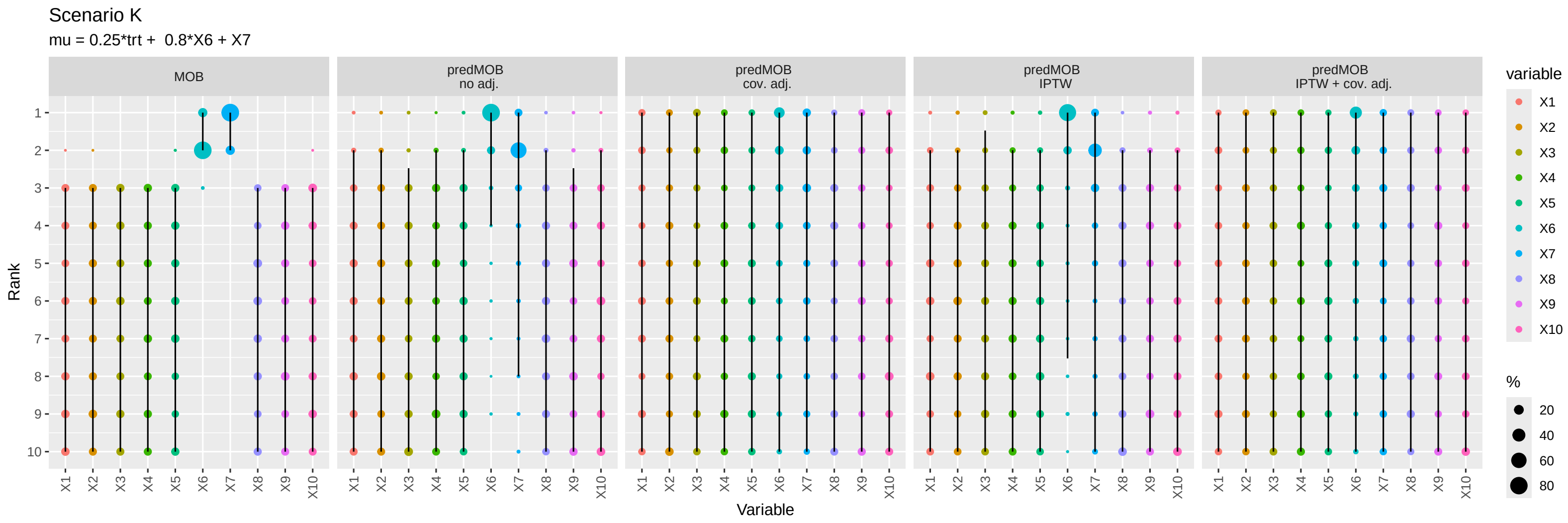
Scenario K
mu = 0.25*trt + 0.8*X6 + X7
MOB
predMOB no adj.
predMOB cov. adj.
predMOB IPTW
predMOB IPTW + cov. adj.
Rank
Variable
variable
X1
X2
X3
X4
X5
X6
X7
X8
X9
X10
%
20
40
60
80

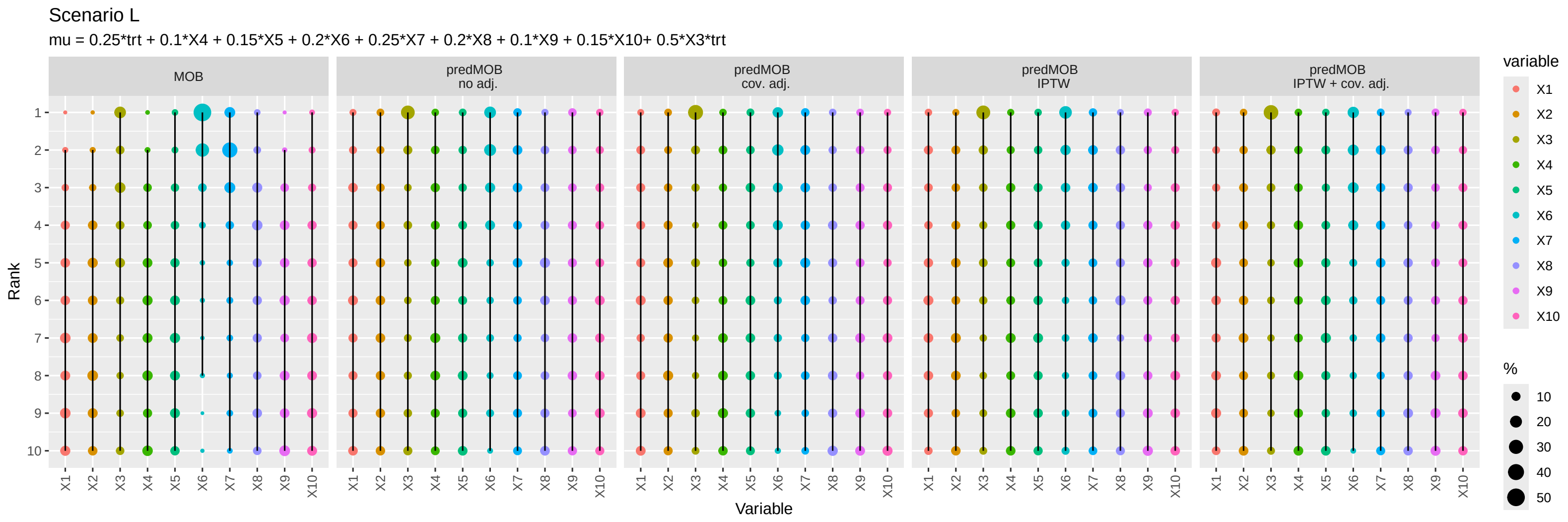
Scenario L
mu = 0.25*trt + 0.1*X4 + 0.15*X5 + 0.2*X6 + 0.25*X7 + 0.2*X8 + 0.1*X9 + 0.15*X10+ 0.5*X3*trt
MOB
predMOB no adj.
predMOB cov. adj.
predMOB IPTW
predMOB IPTW + cov. adj.
Rank
Variable
variable
X1
X2
X3
X4
X5
X6
X7
X8
X9
X10
%
10
20
30
40
50

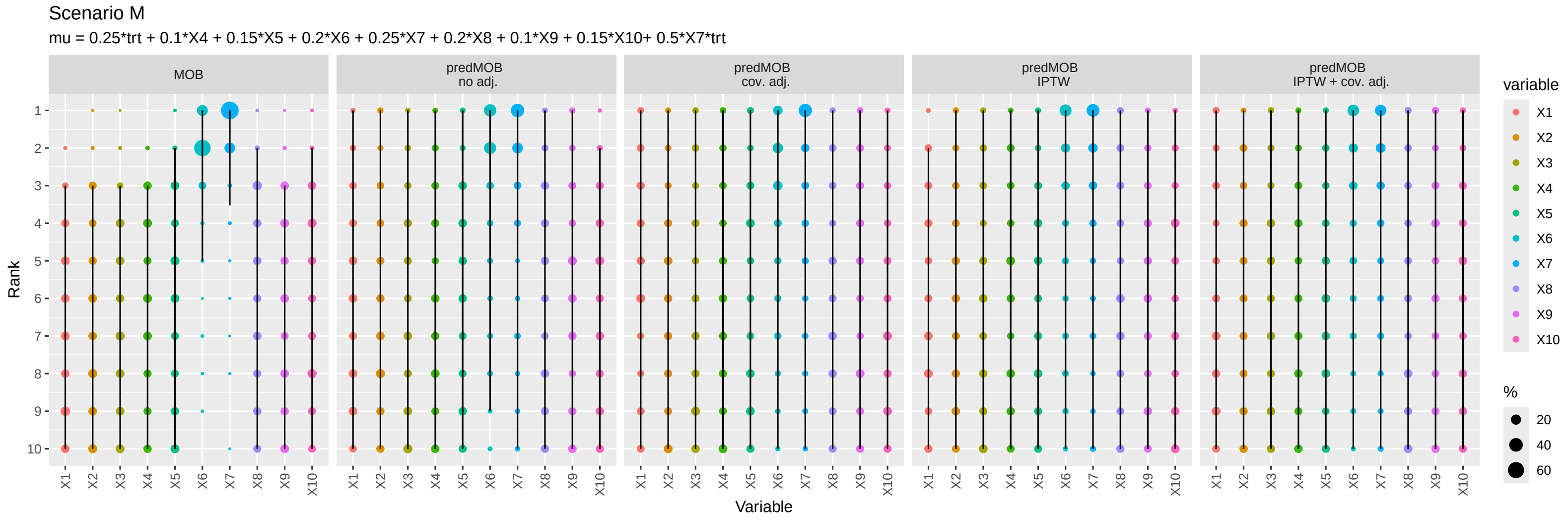

Scenario M
mu = 0.25*trt + 0.1*X4 + 0.15*X5 + 0.2*X6 + 0.25*X7 + 0.2*X8 + 0.1*X9 + 0.15*X10+ 0.5*X7*trt
MOB
predMOB no adj.
predMOB cov. adj.
predMOB IPTW
predMOB IPTW + cov. adj.
Rank
Variable
variable
X1
X2
X3
X4
X5
X6
X7
X8
X9
X10
%
20
40
60

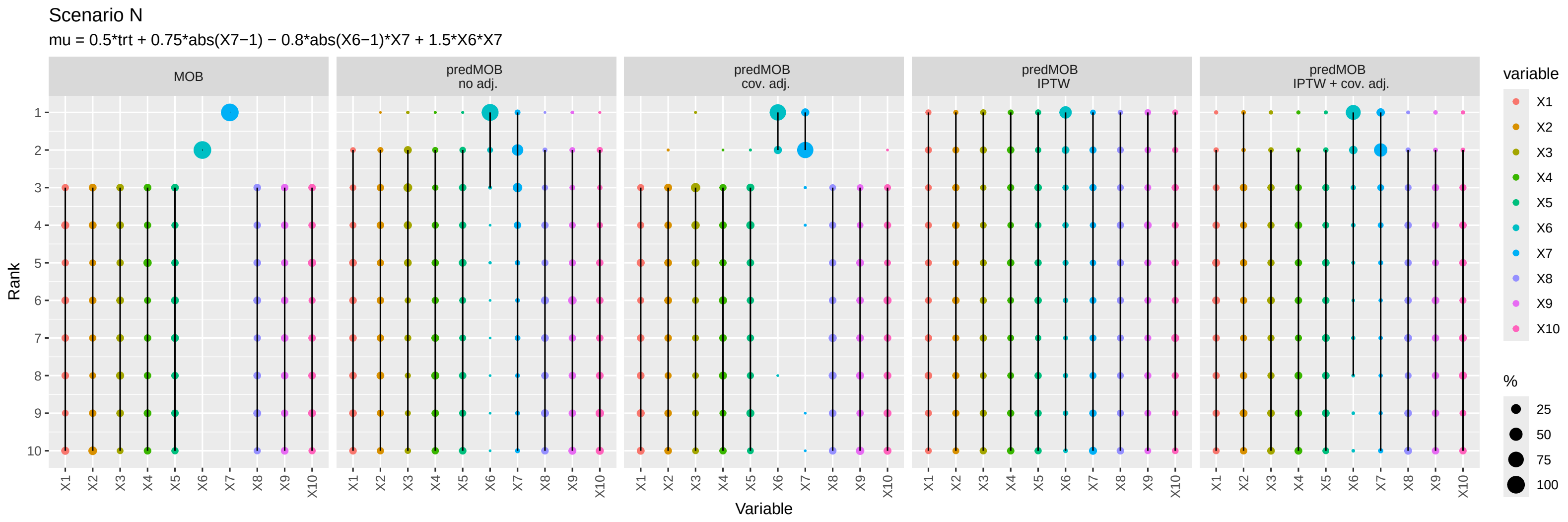
Scenario N
mu = 0.5*trt + 0.75*abs(X7−1) − 0.8*abs(X6−1)*X7 + 1.5*X6*X7
MOB
predMOB
no adj.
predMOB
cov. adj.
predMOB
IPTW
predMOB
IPTW + cov. adj.
Rank
Variable
variable
X1
X2
X3
X4
X5
X6
X7
X8
X9
X10
%
25
50
75
100

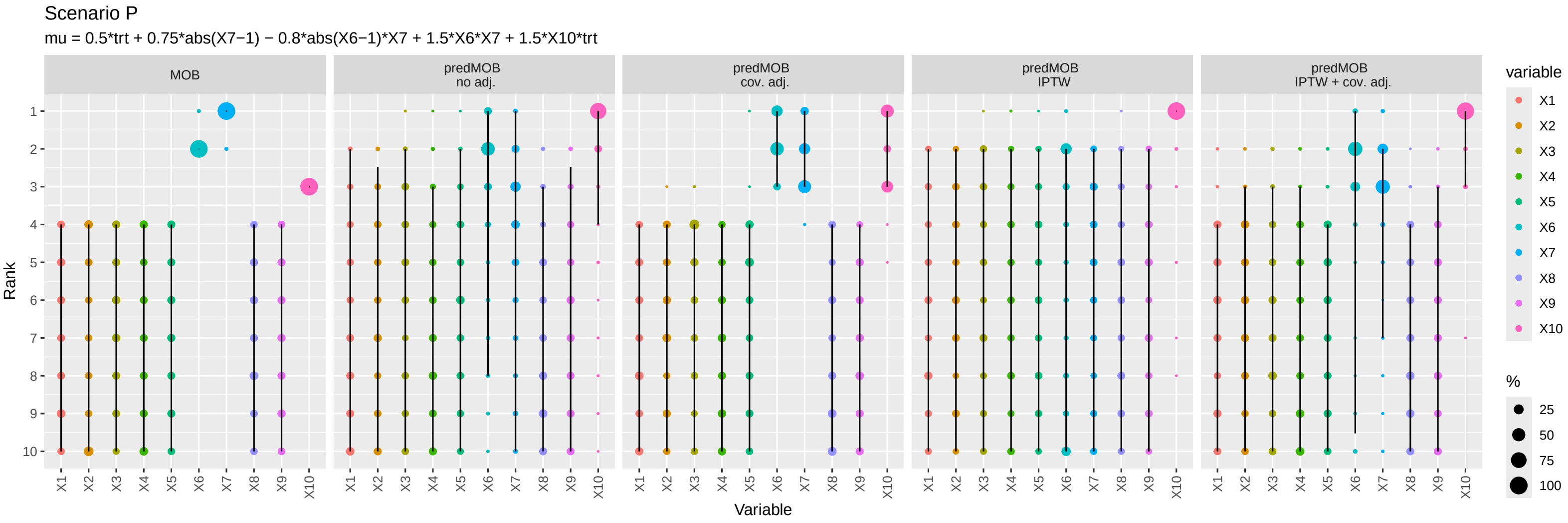
Scenario P
mu = 0.5*trt + 0.75*abs(X7−1) − 0.8*abs(X6−1)*X7 + 1.5*X6*X7 + 1.5*X10*trt
MOB
predMOB no adj.
predMOB cov. adj.
predMOB IPTW
predMOB IPTW + cov. adj.
Rank
Variable
variable
X1
X2
X3
X4
X5
X6
X7
X8
X9
X10
%
25
50
75
100

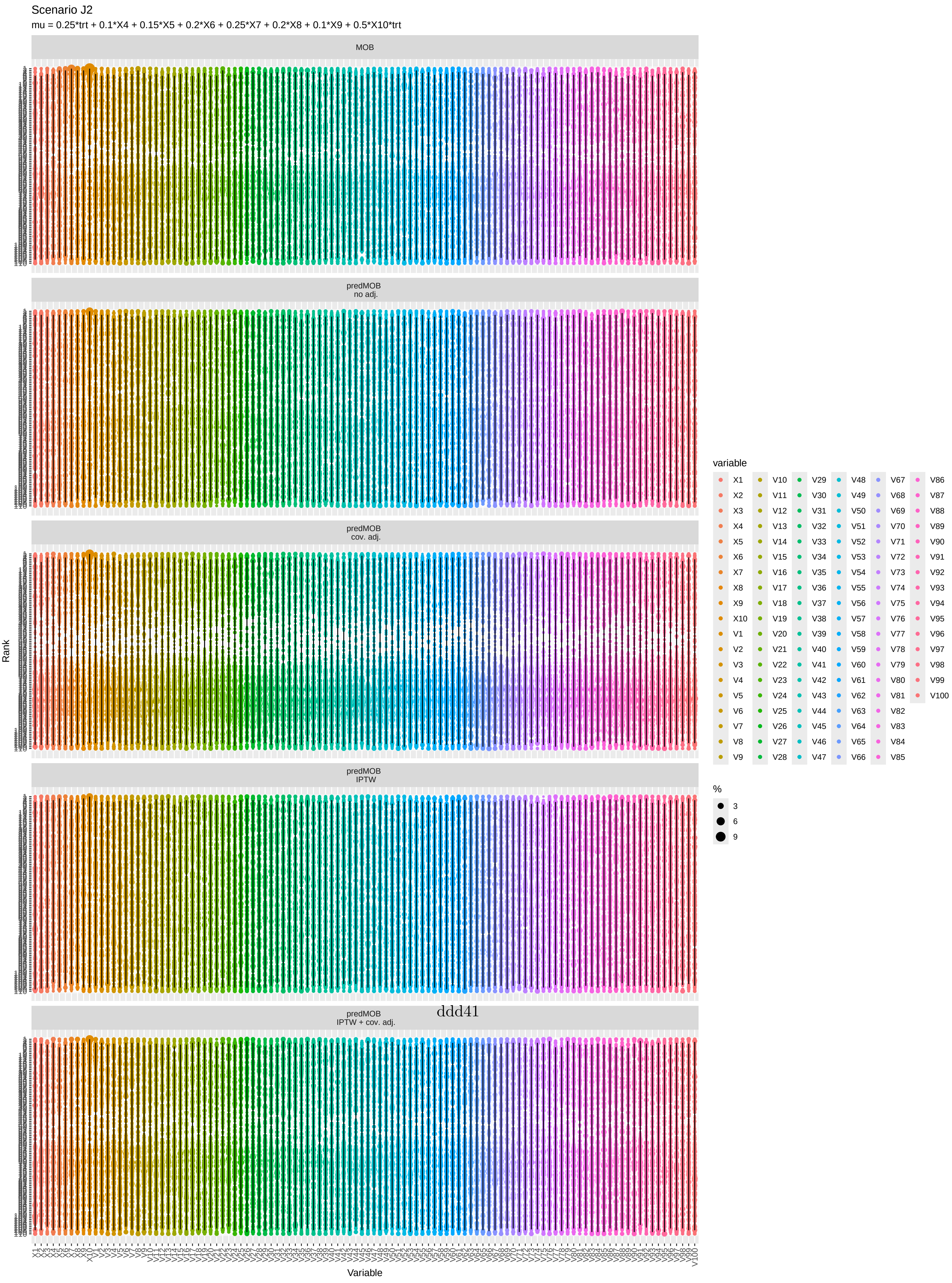
Scenario J2
mu = 0.25*trt + 0.1*X4 + 0.15*X5 + 0.2*X6 + 0.25*X7 + 0.2*X8 + 0.1*X9 + 0.5*X10*trt
MOB
predMOB
no adj.
predMOB
cov. adj.
predMOB
IPTW
predMOB
IPTW + cov. adj.
Rank
Variable
variable
%
3
6
9

S 6.2 Application examples

In this section we present the ranking approach to the results of MOB and predMOB for two clinical applications.

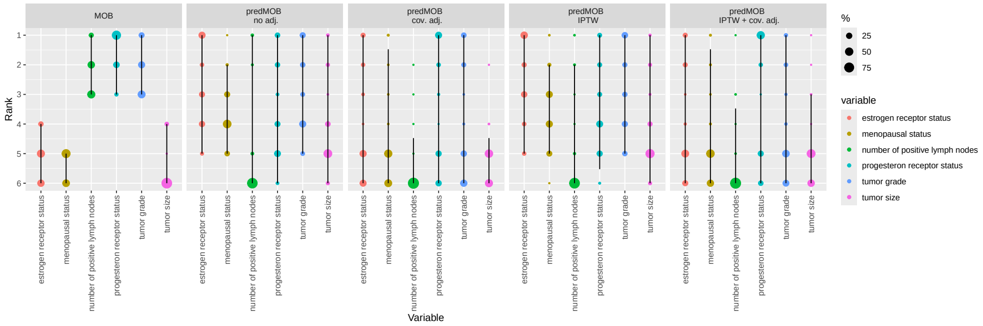

Fig. S.18: In randomized cohort of the GBSG2 trial the ranking of the permutation importance varies across the predMOB approaches.

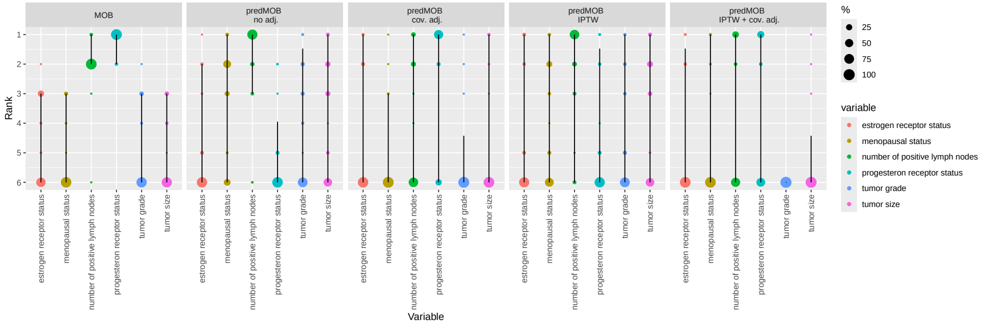

Fig. S.19: In non-randomized cohort of the GBSG2 trial all predMOB approaches assign high permutation importance rank to number of positive lymph nodes. After adjustment for covariates and IPTW this variable shares top rank along with progesterone receptor status.

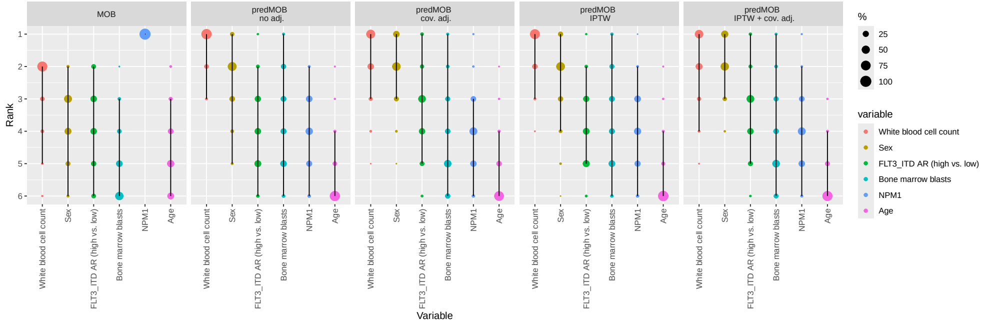

Fig. S.20: Permutation importance ranking of the different variables using various predMOB approaches show that white blood cell count and sex are consistently ranked first and second, indicating possible predictive effects of these variables in the AMLSG 16-10 trial.

S 7 Distribution of covariates by treatment across datasets

In order to give a better overview of how the data generating process described in the simulation study affects the covariate distribution in the two treatment arms, the proportion of observed mutations is plotted by arm for each variable averaged over 500 simulation runs. As mentioned in Section 3 the treatment variable $T \sim B(1, p)$, depends on biomarkers $X_1, \ldots, X_7$ via the logistic regression (propensity score) model $\text{logit}(p) = \beta_0 + \log(1.1)X_1 - \log(1.2)X_2 + \log(1.3)X_3 - \log(1.1)X_4 + \log(1.2)X_5 - \log(1.3)X_6 + \log(1.4)X_7$, with β_0 being chosen so that $p = 0.5$.

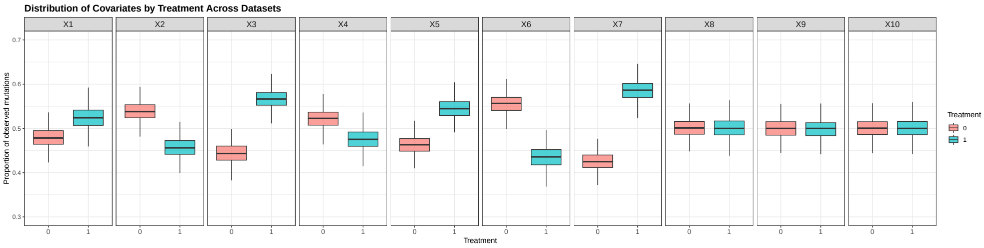

Fig. S.21: Distribution of covariates by treatment averaged over 500 simulated data sets depicting differences in observed mutation frequencies by treatment arms for variables $X_1, \ldots, X_7$ in the same direction and magnitude as in the propensity score model.
